# New insights into the classification of the RAC1 P29S hotspot mutation in melanoma as an oncogene

**DOI:** 10.1038/s41417-025-00965-x

**Published:** 2025-10-01

**Authors:** Amin Mirzaiebadizi, Mohammad Reza Ahmadian

**Affiliations:** Institute of Biochemistry and Molecular Biology II, Medical Faculty and University Hospital Düsseldorf, Heinrich Heine University, Düsseldorf, Germany

**Keywords:** Cancer models, Oncogenes

## Abstract

The RAC1^P29S^ hotspot mutation, which is prevalent in melanoma, drives tumorigenesis by promoting the persistent activation of RAC1. This mutation enhances molecular interactions, and hyperactivates key signaling pathways, making RAC1^P29S^ a promising target for cancer therapy. This study provides a comprehensive biochemical and cell-based characterization of RAC1^P29S^, as well as comparisons with wild-type RAC1 and the T17N and F28L mutants. The P29S substitution significantly impairs nucleotide binding while accelerating intrinsic nucleotide exchange. While it minimally affects regulation by guanosine dissociation inhibitor 1 (GDI1), RAC1^P29S^ exhibits reduced activation via DBL family guanine nucleotide exchange factors (GEFs) but retains effective activation by dedicator of cytokinesis 2 (DOCK2). Importantly, the P29S mutation severely impairs GTPase-activating protein-stimulated GTP hydrolysis, which most likely contributes to RAC1^P29S^ hyperactivation by prolonging its GTP-bound active form. This mutation displays a stronger binding affinity for the IQ motif-containing GTPase-activating protein 1 (IQGAP1) than for the p21-activated kinase 1 (PAK1), indicating altered effector interactions that modulate downstream signaling spatially. These biochemical findings are consistent with the fact that RAC1^P29S^ predominantly adopts an active GTP-bound state under serum-starved conditions. IGR1 human melanoma cells harboring endogenous RAC1^P29S^ exhibit persistent RAC1^P29S^•GTP accumulation, even without upstream GEF activation. Furthermore, the pharmacological inhibition of DOCK2 with CPYPP significantly reduces RAC1^P29S^ activation in these cells, which confirms the pivotal role of DOCK2 in sustaining RAC1^P29S^-driven signaling. Overexpression of RAC1^P29S^ activates key oncogenic pathways, including ERK1/2 and p38 MAPK, highlighting its role as a constitutively active driver mutation. Together, these results imply that targeting upstream regulators such as DOCK2 and downstream effectors, such as IQGAP1, could be effective therapeutic strategies for counteracting RAC1^P29S^-mediated melanoma progression and resistance to targeted therapies.

**Figure:**
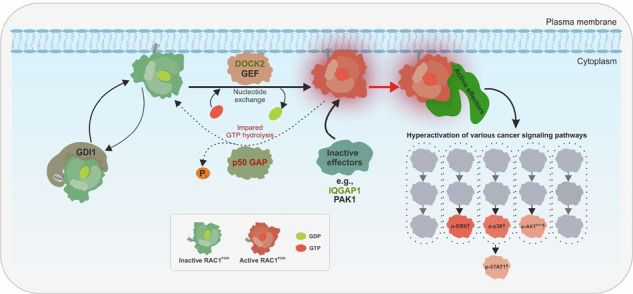
A model of RAC1^P29S^ activation and signaling in cancer cells. RAC1^P29S^ remains in an inactive GDP-bound state in the cytoplasm where GDI1 prevents its membrane association. Upon stimulation, GEFs, primarily DOCK2, activate RAC1^P29S^ by promoting GDP-GTP exchange, facilitating its transition to the active GTP-bound state and initiating downstream signaling. RAC1^P29S^ binds preferentially to IQGAP1 over PAK1, reflecting a shift in effector interactions. IQGAP1 acts as a scaffolding protein, spatially modulating RAC1^P29S^-driven signaling and amplifying its effects. Under normal conditions, GAPs such as p50GAP regulate RAC1 by accelerating GTP hydrolysis, thereby maintaining its dynamic activation cycle. However, the P29S mutation severely impairs p50GAP-mediated hydrolysis, leading to accumulation of RAC1^P29S^ in its GTP-bound state and loss of temporal regulation. This persistent activation hyperactivates downstream effectors and promotes cancer-associated pathways, including ERK and p38 MAPK, which drive cell growth, survival, invasion and metastasis.

A model of RAC1^P29S^ activation and signaling in cancer cells. RAC1^P29S^ remains in an inactive GDP-bound state in the cytoplasm where GDI1 prevents its membrane association. Upon stimulation, GEFs, primarily DOCK2, activate RAC1^P29S^ by promoting GDP-GTP exchange, facilitating its transition to the active GTP-bound state and initiating downstream signaling. RAC1^P29S^ binds preferentially to IQGAP1 over PAK1, reflecting a shift in effector interactions. IQGAP1 acts as a scaffolding protein, spatially modulating RAC1^P29S^-driven signaling and amplifying its effects. Under normal conditions, GAPs such as p50GAP regulate RAC1 by accelerating GTP hydrolysis, thereby maintaining its dynamic activation cycle. However, the P29S mutation severely impairs p50GAP-mediated hydrolysis, leading to accumulation of RAC1^P29S^ in its GTP-bound state and loss of temporal regulation. This persistent activation hyperactivates downstream effectors and promotes cancer-associated pathways, including ERK and p38 MAPK, which drive cell growth, survival, invasion and metastasis.

## Introduction

As a key member of the RHO guanosine triphosphatase (GTPase) family, Ras-related C3 botulinum toxin substrate 1 (RAC1) functions as a molecular switch, cycling between an inactive guanosine diphosphate (GDP)-bound form and an active guanosine triphosphate (GTP)-bound form [1]. This switch relies on two essential processes: GDP/GTP exchange and GTP hydrolysis, which induce structural changes in the switch I (amino acids 29–42) and switch II (amino acids 62–68) regions [2]. These functions are regulated by guanine nucleotide exchange factors (GEFs) and GTPase-activating proteins (GAPs) [3–5]. The RHO GEF family includes the structurally distinct dedicator of cytokinesis (DOCK) and diffuse B-cell lymphoma (DBL) subfamilies [1, 6, 7]. In addition, guanine nucleotide dissociation inhibitors (GDIs) selectively bind geranylgeranylated RAC1, controlling its membrane localization [8].

RAC1 and its isoform RAC1B [9] and paralogs RAC2 and RAC3 [10] activate diverse signaling pathways through direct interaction with effector proteins [1]. These interactions regulate essential cellular processes, including motility, oxidative stress, and inflammation [11]. GTP-bound RAC1 binds effectors, activating kinases like p21-activated kinase 1 (PAK1) and scaffolding proteins like IQ motif-containing GTPase-activating protein 1 (IQGAP1) [1]. Dysregulation [12, 13] or gain-of-function mutations in RAC genes [14, 15] can hyperactivate RAC signaling, altering cellular responses and contributing to cancer. This dysregulation contributes to various pathological conditions, including cancer [16], and other pathological conditions, including metabolic, neurodegenerative, cardiovascular, inflammatory, and infectious diseases [11].

The proline 29 to serine (P29S) mutation in RAC1 is the third most common hotspot mutation in melanoma, following BRAF V600E and neuroblastoma RAS viral oncogene homolog (NRAS) Q61R [17]. Despite its prevalence, the regulatory functions driving RAC1^P29S^ pro-tumorigenic effects remain poorly understood [18]. Functional studies show that RAC1^P29S^ enhances effector binding, including PAK1 and mixed lineage kinase 3 (MLK3), promoting melanocyte proliferation and migration [19, 20]. Additionally, RAC1^P29S^ inhibits invadopodia function [21], abolishes haptotaxis [22], drives dedifferentiation in melanoma, contributes to BRAF inhibitor resistance [23–25], and facilitates immune evasion via programmed death-ligand 1 (PD-L1) upregulation through the RAC1^P29S^-PAK1 axis [17]. This immune evasion is mediated by the RAC1^P29S^-PAK1 axis, which promotes the G2/M cell cycle transition through phosphorylation of Aurora kinase A and polo-like kinase 1 (PLK1) [26] and inactivates neurofibromin 2 (NF2)/Merlin, promoting proliferation, metastasis, and drug resistance [27]. Furthermore, while BRAF^V600E^ suppresses cell migration, extracellular signal-regulated kinase (ERK) pathway inhibition accelerates migration and invasion in BRAF^V600E^- and mutant RAS-driven tumors [28]. Although RAC1 is a critical therapeutic target in melanoma, its undruggable nature poses a significant challenge for targeting RAC1^P29S^ [11, 29–35].

Initial studies using radiolabeled nucleotide filter binding assays or thin-layer chromatography compared the basal GDP/GTP exchange and GTP hydrolysis of RAC1^P29S^ with RAC1^WT^. Davis et al. reported increased GTP dissociation for RAC1^P29S^ [36], while Kawazu et al. observed increased GDP dissociation but not GTP dissociation [37]. Both studies concluded that GTP hydrolysis remained unchanged. However, these and other overexpression studies alone cannot classify RAC1^P29S^ as spontaneously activating, self-activating, fast cycling, constitutively active, or oncogenic (Box 1) [20, 21, 36, 38, 39]. Some of these classifications are derived from assumptions about the phenylalanine 28 to leucine (F28L) mutant of RAC1. Although RAC1^F28L^ is not extensively studied, it is described as a fast-cycling mutant, analogous to CDC42^F28L^, capable of spontaneous nucleotide exchange without GEF activation while retaining full GTPase activity [40]. Another widely studied mutant, threonine 17 to aspargine (T17N), is a dominant negative mutant with T17 in the phosphate-binding loop (P-loop), a region critical for nucleotide binding, while F28 and P29 reside at the N-terminus of switch I. The P-loop and switch I are essential for RAC1 nucleotide binding and hydrolysis [1]. Biophysical and biochemical studies, supported by molecular dynamics simulations, indicate that the P29S mutation increases switch I flexibility, adopting an open conformation that facilitates rapid GDP/GTP exchange in RAC1 [20, 36, 41, 42].

This study provides a comprehensive characterization of RAC1^P29S^ at three levels: intrinsic properties, regulation, and effector interaction. At the intrinsic level, we analyzed its nucleotide exchange kinetics, GTP hydrolysis capacity, and binding affinities for GDP and GTP. We assessed regulatory mechanisms by examining activation via DBL and DOCK family GEFs, as well as p50 Rho GTPase-activating protein (p50GAP)-mediated GTP hydrolysis and GDI1-mediated regulation. Effector interactions were evaluated using PAK1, a representative kinase, and IQGAP1, a scaffolding protein that spatially modulates RAC1 signaling. Active GTPase pull-down assays performed under serum-stimulated and serum-starved conditions provided further insights into the cellular GTP-bound state of RAC1^P29S^. Comparative analyses with RAC1^WT^, RAC1^T17N^, and RAC1^F28L^ revealed distinct biochemical features of RAC1^P29S^, including an accelerated intrinsic nucleotide exchange rate, preferential activation by DOCK2, and severely impaired p50GAP-stimulated GTP hydrolysis. Consistent with these findings, we demonstrated that RAC1^P29S^ remains predominantly GTP-bound in IGR1 human melanoma cells, even under serum-starved conditions. Furthermore, we showed that pharmacological inhibition of DOCK2 using CPYPP significantly reduces its activation. These results confirm the pathological persistence of active RAC1^P29S^ in melanoma and reinforce the critical role of DOCK2 in maintaining its oncogenic signaling. Taken together, our findings identify RAC1^P29S^ as a constitutively active mutant and highlight DOCK2, p50GAP, and IQGAP1 as potential therapeutic targets for suppressing RAC1^P29S^-driven melanoma progression.

Box 1 The terminologies of the mutations and their effects on the intracellular regulation and function of small GTPases, using the example of RAC1 **• Dominant negative mutations** impair nucleotide binding affinity to the extent that RAC1 forms a non-functional complex with its cognate GEFs in a nucleotide-free state. A dominant negative RAC1 prevents the activation of wild-type RAC1 when both are present in the same cell, leading to a loss of RAC1 activity and disruption of its downstream signaling pathways.

**• Spontaneous activation mutations** affect the basal activities of RAC1, including enhanced GDP/GTP exchange and reduced GTP hydrolysis. A spontaneously activated RAC1 bypasses normal regulatory mechanisms (without typical regulatory input from other cellular components) and initiates its function independently, leading to unregulated signaling and potentially contributing to cellular dysfunction and disease.

**• Self-activating mutations** refer to the ability of RAC1 to autonomously initiate its signaling function without requiring the usual activation by other cellular components or regulatory proteins. A self-activating RAC1 spontaneously binds GTP and hydrolyzes it to GDP without external regulatory input. This autonomous activation can lead to uncontrolled signaling pathways, potentially contributing to cellular dysfunctions and diseases such as cancer.

**• Fast cycling mutations** lead to the rapid turnover rates of the GDP/GTP exchange and GTP hydrolysis of RAC1. A fast-cycling GTPase rapidly cycles between the inactive, GDP-bound state and the active, GTP-bound state, allowing for quick and dynamic regulation of cellular processes.

**• Constitutively active mutations** affect the GTP hydrolysis reaction of RAC1, resulting in an increased proportion of its active, GTP-bound state, regardless of cellular signaling cues. A constitutively active RAC1 continuously promotes downstream signaling pathways, even when the cell is quiescent or upstream signaling is blocked.

**• Oncogenic mutations** lead to overactivation/hyperactivation of RAC1 and drive oncogenesis. The accumulation of oncogenic RAC1 in its GTP-bound state leads to uncontrolled cellular activities that may contribute to the initiation and progression of various types of cancer.

## Material and methods

### Constructs

Human RAC1 wild-type (RAC1^WT^; accession no. P63000) and its mutants T17N, F28L, and P29S were expressed as N-terminal glutathione S-transferase (GST)-tagged fusion proteins using pGEX vectors (pGEX-2T and pGEX-4T-1). The same system was used to express regulators and effectors, including full-length GDI1, the Dbl homology-pleckstrin homology (DH-PH) tandem domains of T-lymphoma invasion and metastasis-inducing protein 1 (TIAM1), vav guanine nucleotide exchange factor 2 (VAV2), son of sevenless homolog 1 (SOS1), and phosphatidylinositol-3,4,5-trisphosphate-dependent Rac exchanger 1 (PREX1); the GAP domain of p50GAP; the C-terminal 794-amino acid region of IQGAP1; and the RAC1 binding domain (RBD) of PAK1. Additional constructs included His-tagged IQGAP1 (pET-23b+ vector) and His6-small ubiquitin-like modifier (SUMO)-tagged DOCK2 Dock homology region 2 (DHR2) domain (pOPINS vector). RAC1 constructs with N-terminal tandem decahistidine triple-flag tags were cloned into the pcDNA-3.1 vector for eukaryotic expression. Detailed constructs descriptions, including accession numbers and amino acid sequences, are available in the Supplementary information.

### Proteins

All proteins were purified as described previously [3, 5, 9, 10, 43]. Briefly, Escherichia coli strains were transformed for protein expression, lysed, and subjected to affinity purification using GST or His tags. GST tags were cleaved when necessary, and proteins were buffer-exchanged into optimized storage buffers. Purity was confirmed by SDS-PAGE and Coomassie staining (Supplementary Fig. S1), which shows the purified proteins used in this study. Proteins were stored at −80 °C. Detailed procedures are available in the Supplementary Materials.

### Preparation of nucleotide-free and fluorescent nucleotide-bound GTPases

As previously described, nucleotide-free GTPases were prepared through sequential treatment with alkaline phosphatase and snake venom phosphodiesterase [44, 45]. Fluorescent GDP- and GppNHp-bound GTPases were generated by incubating nucleotide-free proteins with mant-labeled nucleotides (mdGDP and mGppNHp). Protein concentrations were quantified by high-performance liquid chromatography (HPLC) after buffer exchange using NAP-5 columns. Samples were stored at −80 °C. Detailed procedures are provided in the Supplementary Materials.

### Fluorescence kinetic measurements

Fluorescence-based kinetic measurements for long-term and rapid reactions were performed using a Horiba Fluoromax-4 fluorimeter and a stopped-flow spectrophotometer (Applied Photophysics SX20), as described [43–46]. Excitation and emission wavelengths were set according to the fluorophore-specific properties of mant- and tamra-labeled nucleotides. Detailed experimental conditions are provided in the Supplementary Materials.

### Nucleotide-binding assay

The nucleotide-binding properties of RAC1 GTPases were assessed by stopped-flow fluorimetry, as described [47]. Nucleotide association and dissociation rates were measured using fluorescent nucleotides (mdGDP and mGppNHp) and varying RAC1 concentrations. Association (*k*_on_) and dissociation (*k*_off_) rate constants were determined, and equilibrium dissociation constants (*K*_d_) were calculated as described in Box 2. Detailed procedures are provided in the Supplementary Materials.

Box 2 The definition of various kinetic and equilibrium constants in the context of protein-ligand or protein-protein interactions are described as fallow **• Observed rate constants**: The observed rate constant (***k***_**obs**_) reflects the overall rate at which the interaction occurs, taking into account both the association of the proteins or the protein and ligand, as well as any potential subsequent reactions such as conformational changes or product formation. This is often used in cases where the binding is not at equilibrium and may vary with the concentration of the interacting partners.

**• Association rate constant**: The association rate constant (***k***_**on**_) measures the rate at which a protein and a ligand or two proteins come together to interact with each other and form a complex. It is defined as: *k*_on_ = [PL]/[P][L], Where [PL] is the concentration of the protein-ligand or protein-protein complex, and [P] and [L] are the concentration of free protein and free ligand. A higher *k*_on_ indicates a faster rate of complex formation.

**• Dissociation rate constant**: The dissociation rate constant (***k***_**off**_) quantifies how quickly the protein-ligand or protein-protein complex dissociates back into the free components. This constant is important in determining the stability of the interaction; a higher k_off_ indicates a less stable complex. It can be measured experimentally by monitoring the concentration of the complex or over time after dilution or removal of the ligand.

**• Catalytic rate constant**: In the context of enzyme-ligand interactions, the catalytic rate constant (**k**_**cat**_) refers to the maximum rate of product formation for an enzyme when it is saturated with substrate. For protein-protein interactions, this term may not apply unless there is a specific enzymatic function associated with the interaction, such as in signaling complexes. This constant indicates how efficiently the enzyme catalyzes the reaction after the binding event.

**• Dissociation constants**: The dissociation constant (***K***_**d**_) is critical for understanding the affinity between a protein and its ligand or between two interacting proteins. It is defined as *K*_d_ = *k*_off_/*k*_on_. A lower *K*_d_ indicates a higher affinity between the protein and its partner, meaning they bind more tightly. It is often used to assess the strength of the interaction and is expressed in molar concentration units (M).

**• Equilibrium dissociation constants**: The equilibrium dissociation constant (e**K**_**d**_) measures the affinity between a protein and a ligand or between two proteins in a more complex or biochemical context. It represents the equilibrium state of a reversible binding interaction and is defined as the ratio of the rate constants of dissociation and association. This constant is particularly relevant when considering interactions that occur in environments where factors such as concentration, binding site availability, or the presence of other interacting partners may influence the overall binding dynamics. In a binding reaction where a ligand (L) binds to a protein (P) to form a complex (PL), It is given by the formula eK_d_ = [R][L]/[RL]. A low eK_d_ value indicates a high affinity between the proteins and its ligand, meaning they bind tightly, while a high eK_d_ value indicates low affinity, meaning they bind weakly.

### GEF-catalyzed nucleotide dissociation assay

The GEF-catalyzed nucleotide exchange reaction was monitored by stopped-flow fluorimetry, as described [45]. Reactions were performed with mGDP-bound RAC1 and excess non-fluorescent nucleotide in the presence of GEFs from the DBL and DOCK families. Observed rate constants were analyzed using a single-exponential model in Origin software. Detailed procedures are provided in the Supplementary Materials.

### Intrinsic and GAP-stimulated GTP-hydrolysis assays

The intrinsic GTP hydrolysis rate of RAC1 proteins was determined by HPLC, as described [45]. Reactions were performed with nucleotide-free RAC1 and GTP in a GAP buffer at 25 °C, and catalytic rate constants (*k*_cat_) were calculated using Origin software. GAP-stimulated hydrolysis rates were measured by stopped-flow fluorimetry using tamra-GTP, as described [48]. Detailed procedures are provided in the Supplementary Materials.

### Protein-protein interaction kinetics

The interaction of RAC1 with GST-GDI1, GST-PAK1 RBD, and His-IQGAP1 C794 was analyzed by stopped-flow fluorimetry to determine *k*_on_, *k*_off_, and *K*_d_ values, as described [43]. Binding assays were performed using mdGDP- and mGppNHp-bound RAC1 with varying protein concentrations, and rate constants were calculated using linear regression and single-exponential fits. Detailed procedures are provided in the Supplementary Materials.

### Fluorescence polarization

Fluorescence polarization was used to determine the binding affinity between RAC1 and effector proteins, as described [43]. Assays were performed with mGppNHp-bound RAC1 (1 μM) and titrated effectors in buffer containing 30 mM Tris-HCl (pH 7.5), 50 mM NaCl, 5 mM MgCl_2_, and 3 mM DTT at 25 °C. *K*_d_ values were calculated by fitting binding curves to a quadratic ligand binding equation. Detailed procedures are provided in the Supplementary Materials.

### Cell culture, transfection, and treatment

HEK-293T and IGR1 human melanoma cells were cultured under serum-stimulated and serum-starved conditions in DMEM supplemented with 10% FBS and 1% penicillin/streptomycin. The HEK-293T cells were then transfected with RAC1 constructs containing N-terminal 10×His–triple FLAG tags using TurboFect™ (Thermo Fisher Scientific), according to the manufacturer’s protocol. The HEK-293T cells were cultured and harvested under serum-stimulated or serum-starved conditions without additional treatment. IGR1 cells, in contrast, were treated with the DOCK2 inhibitor CPYPP (MedChemExpress) at 25 µM and 100 µM concentrations, with 0.5% DMSO as a vehicle control. After treatment, cells were harvested and lysed, and protein concentrations were determined using the Bradford assay. Detailed protocols, including buffer compositions, are provided in the Supplementary Information.

### In vitro pull-down assays

Pull-down assays were conducted to assess RAC1 binding to PAK1 RBD and IQGAP1 C794. GST-PAK1 RBD and His-IQGAP1 C794 were immobilized on glutathione-agarose and His-Mag Sepharose Ni beads, respectively. GppNHp-bound RAC1 proteins were incubated with the beads, washed, eluted, and analyzed by SDS-PAGE followed by immunoblotting. Detailed protocols are provided in the Supplementary Materials.

### Active GTPase pull-down assay

This assay was performed to evaluate the levels of GTP-bound (active) RAC1 in HEK-293T cells transiently transfected with RAC1 constructs, as well as in IGR1 human melanoma cells that endogenously express the RAC1^P29S^ mutant without transfection. Experiments were conducted under both serum-stimulated and serum-starved conditions, as described previously [49]. GST–PAK1 RBD- and GST–IQGAP1 C794-coupled beads were prepared and incubated with cell lysates from HEK-293T or IGR1 cells. After incubation, the beads were washed and analyzed by SDS-PAGE followed by immunoblotting. Detailed protocols are provided in the Supplementary Materials.

### Antibodies and immunoblotting

Primary and secondary antibodies were diluted in TBST with a blocking buffer. The antibodies included α-RAC1, α-6x-His, α-flag, α-γ-tubulin, α-p-ERK1/2, α-t-ERK1/2, α-p-AKT, α-t-AKT, α-p-p38 MAPK, α-p38 MAPK, α-p-STAT1, α-STAT1, α-GAPDH, and α-GST. Immunoblots were visualized using the Odyssey® XF Imaging System. Detailed antibody lists and protocols are provided in the Supplementary Materials.

### Statistical analysis

Data in bar graphs represent mean ± S.D., with replicate numbers detailed in figure legends. Immunoblot intensities were quantified using Image Studio Lite 5.2. For the in vitro pull-down assays, the data were normalized based on the ratio of RAC1 to effector relative to the input levels. Active RAC1^P29S^•GTP levels in HEK-293T cells were calculated using bead-bound GST-effector normalization and were adjusted according to the Flag-RAC1/γ-tubulin ratio. In IGR1 cells, active RAC1^P29S^•GTP levels were normalized to bait protein levels and to the endogenous RAC1^P29S^/GAPDH ratio. Downstream signaling data were normalized to phospho/total protein ratios and further adjusted to GAPDH, with Flag-RAC1 levels excluded from normalization to avoid bias. Statistical significance was determined using one-way ANOVA followed by Tukey’s test (*P ≤ 0.05, **P ≤ 0.01, ***P ≤ 0.001, ****P ≤ 0.0001). Detailed normalization methods and calculations are provided in the Supplementary Materials.

## Results

### P29S significantly impairs the nucleotide binding of RAC1

**Fig. 1:**
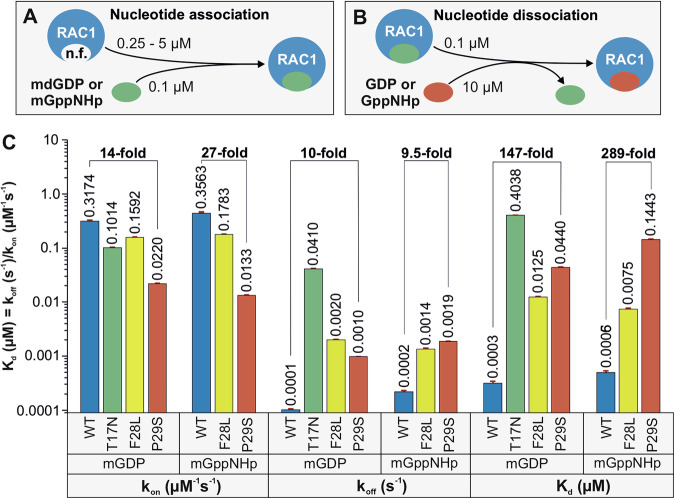
Severe impairment of the GDP/GTP binding properties of RAC1^P29S^. The kinetics of association (**A**) and dissociation (**B**) of fluorescent mdGDP and mGppNHp with RAC1 proteins were measured as illustrated. **C** Kinetic rate constants for association (*k*_on_) and dissociation (*k*_off_), as well as the dissociation constant (*K*_d_), calculated from the *k*_off_/*k*_on_ ratio, reveal substantial effects of the P29S mutation on the binding of mdGDP and mGppNHp to RAC1. These effects differ markedly from those observed for the T17N and F28L substitutions. This impaired binding may contribute to the accelerated intrinsic nucleotide exchange observed in RAC1^P29S^. All *k*_on_, *k*_off_, and *K*_d_ values, presented as bar graphs, represent the average of three to six measurements and are reported as means ± SD.

Two sets of real-time kinetic measurements were performed to investigate the impact of the P29S mutation on nucleotide binding affinity. The first measured the association of mdGDP and mGppNHp with nucleotide-free (n.f.) RAC1 (Fig. 1A), while the second analyzed the dissociation of these nucleotides from RAC1 (Fig. 1B). The fluorescent analog mdGDP was used as a substitute for GDP, and the non-hydrolyzable mGppNHp replaced GTP. The RAC1 variants included WT, T17N, F28L, and P29S.

Binding of nucleotides to n.f. RAC1 induced a rapid fluorescence increase, with *k*_obs_ values rising proportionally with n.f. RAC1 concentrations (supplementary Figs. S2 and S3, left panels), which depict the interaction of mdGDP and mGppNHp with RAC1 at increasing concentrations. The *k*_on_ values for mdGDP and mGppNHp binding were derived from linear fits of *k*_obs_ values across protein concentrations (Supplementary Figs. S2 and S3, middle panels), where k_on was determined by plotting observed rate constants from exponential fits of association data against the corresponding RAC1 concentrations. A bar graph of *k*_on_ values showed significant differences in nucleotide association among RAC1 variants (Fig. 1C). The P29S mutation notably reduced the association of mdGDP and mGppNHp with RAC1 by 14-fold and 27-fold, respectively, compared to RAC1^WT^.

A decrease in fluorescence was observed during nucleotide dissociation from RAC1 proteins in the presence of excess free GDP (Supplementary Figs. S2 and S3, right panels), which depict the dissociation kinetics of mdGDP and mGppNHp from RAC1 proteins. The *k*_off_ values, derived from single exponential fits of the dissociation data, are shown as bar graphs (Fig. 1C). RAC1^P29S^ and RAC1^F28L^ exhibited intrinsic nucleotide dissociation rates 10- and 20-fold faster than RAC1^WT^, respectively. RAC1^T17N^ showed the fastest mdGDP dissociation rate, 410-fold higher than RAC1^WT^, resulting in a significantly reduced *K*_d_ and a 1346-fold decrease in binding affinity, highlighting its dominant-negative effect (see Box 1 for Definitions). Additionally, due to extremely rapid association and dissociation rates, mGppNHp kinetics for RAC1^T17N^ could not be determined using stopped-flow fluorimetry. This was further confirmed by fluorimeter-based measurements, which demonstrated its rapid nucleotide exchange properties (Supplementary Fig. S3, lower panel).

Nucleotide-binding affinity (*K*_d_) was calculated using kinetic parameters for dissociation and association reactions. RAC1^WT^ displayed tight binding affinities for mdGDP and mGppNHp, with *K*_d_ values of 0.3 nM and 0.6 nM, respectively. These affinities were significantly reduced for RAC1^T17N^, followed by RAC1^P29S^ and RAC1^F28L^ (Fig. 1C). Due to rapid kinetics, the mGppNHp binding affinity for RAC1^T17N^ could not be determined (Supplementary Fig. S3, lower panel), where fluorescence measurements demonstrated its inability to be analyzed via standard stopped-flow techniques. RAC1^P29S^ showed markedly impaired nucleotide binding, with 147-fold and 289-fold lower affinities for mdGDP and mGppNHp, respectively. These findings suggest that RAC1^P29S^’s impaired binding properties likely drive its accelerated intrinsic nucleotide exchange, although further structural studies on its interactions with regulators and effectors are needed to elucidate its aberrant behavior.

### Only the T17N mutation significantly impairs GDI1 activity

**Fig. 2:**
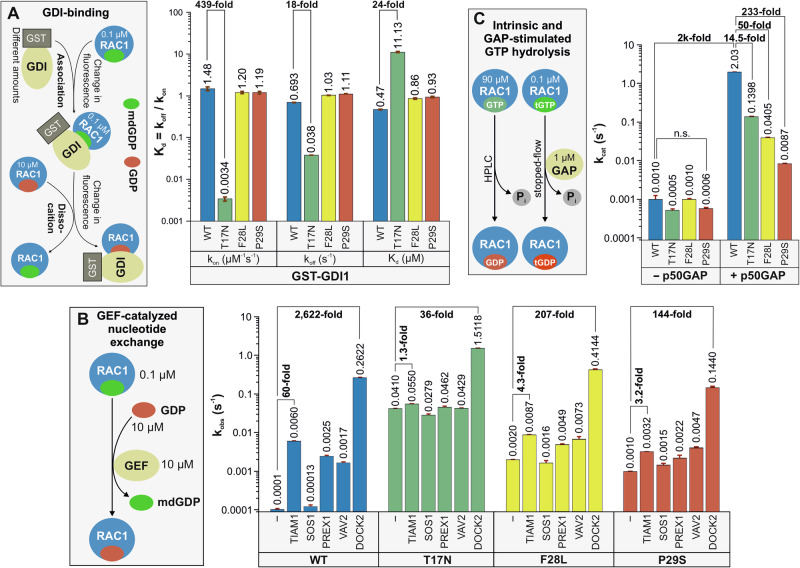
Effects of mutations on the regulation of RAC1 by GDI1, various GEFs, and p50GAP. **A** Minimal effect of the P29S mutation on the RAC1-GDI1 interaction. The principle behind the kinetic measurements of the association of GST-GDI1 with RAC1 proteins and its dissociation is illustrated using a stopped-flow instrument. In these experiments, 0.1 µM mdGDP-bound RAC1 was rapidly mixed with increasing concentrations of GST-GDI1 to monitor the association kinetics. Dissociation kinetics were measured by rapidly mixing a complex of RAC1•mdGDP•GST-GDI1 with excess GDP-bound RAC1. Bar graphs from the stopped-flow analysis depict the association rates (*k*_on_) and dissociation rates (*k*_off_) of the GDI1 interaction from/with RAC1 proteins, as well as the dissociation constant (*K*_d_), calculated from the *k*_off_/*k*_on_ ratio. The analysis revealed a substantial reduction in GDI1 binding affinity for RAC1^T17N^ and a slight reduction for RAC1^P29S^. All kinetic data are based on the average of three to six measurements and are presented as mean ± SD. **B** Impairment of the catalyzed nucleotide exchange of RAC1^P29S^ by DBL proteins but not by DOCK2. The mdGDP-to-GDP exchange of RAC1 proteins was measured in the absence and presence of the DH-PH tandem of various DBL family members (TIAM1, SOS1, PREX1, and VAV2) and the DHR2 domain of DOCK2, a member of the DOCK family. The observed rate constants (*k*_obs_), shown as bar graphs, represent the average of three to six measurements and are displayed as means ± SD. **C** Severely impaired GAP-stimulated GTP hydrolysis reaction of RAC1^P29S^. The basal and p50GAP-stimulated GTP hydrolysis reactions were measured using HPLC and stopped-flow instruments, respectively. The determined catalytic rate constants (*k*_cat_), presented as bar graphs, are based on duplicate measurements for HPLC data and three to six measurements for stopped-flow data and are reported as means ± SD.

We recently developed a fluorescence-based method to monitor RAC1-GDI1 interactions [8]. Our results showed that GDI1 binding, essential for GDI-mediated membrane translocation, does not differentiate between non-prenylated and prenylated RAC1. Real-time kinetic measurements evaluated the association and dissociation kinetics of GDI1 with mdGDP-bound RAC1 (Fig. 2A, left panel; Supplementary Fig. S4), which presents the binding of RAC1 to GST-GDI1 across increasing concentrations, followed by kinetic analysis. Corresponding rate constants are shown in Fig. 2A, right panel. RAC1^F28L^ and RAC1^P29S^ exhibited *k*_on_ and *k*_off_ values comparable to RAC1^WT^, with slightly reduced GDI1 binding affinity for RAC1^P29S^. In contrast, RAC1^T17N^ displayed a 439-fold decrease in GDI association and an 18-fold reduction in dissociation, leading to a significantly decreased binding affinity compared to RAC1^WT^.

### RAC1^P29S^ is mainly activated by DOCK2 and not by DBL family GEFs

To assess GEF-mediated nucleotide exchange, we evaluated mdGDP dissociation from RAC1^WT^, RAC1^T17N^, RAC1^F28L^, and RAC1^P29S^ in the presence of various RAC1-selective GEFs, including DBL family members (TIAM1, VAV2, SOS1, PREX1) [3, 4] and DOCK family member DOCK2 [7, 50] (Fig. 2B, left panel; Supplementary Fig. S5), which presents kinetic measurements of GEF-catalyzed mdGDP dissociation from RAC1 proteins. Fluorescence decay curves were fitted to a single exponential function to determine *k*_off_ values in the presence of each GEF. Substantial GEF activity against RAC1^WT^ was observed for the DH-PH domains of TIAM1, PREX1, and VAV2, but not SOS1, consistent with prior reports [3]. This lack of SOS1 activity extended to RAC1 mutants. Our findings indicate that RAC1^P29S^ has slow basal nucleotide exchange with DBL proteins and is primarily activated by DOCK2. The DHR2 domain of DOCK2 exhibited 40-fold greater activity than TIAM1 against RAC1^WT^ and showed significant GEF activity for RAC1^P29S^ and other mutants (Fig. 2B). This evidence positions DOCK2 as the primary potential activator of RAC1^P29S^ in cancer cells, particularly in melanoma.

### The P29S mutation significantly impairs the GAP activity

GTP hydrolysis was evaluated using HPLC for intrinsic hydrolysis and stopped-flow fluorimetry for GAP-stimulated hydrolysis (Fig. 2C; Supplementary Fig. S6), which presents measurements of both basal and GAP-stimulated GTP hydrolysis of RAC1 proteins. Intrinsic hydrolysis was assessed by quantifying relative GTP content via HPLC, while real-time hydrolysis in the presence of p50GAP was analyzed using stopped-flow fluorescence. RAC1^WT^ exhibited slow intrinsic GTP hydrolysis (*k*_cat_ = 0.001 s⁻¹), consistent across RAC1 mutants, including RAC1^P29S^ (Fig. 2C). In contrast, RAC1^P29S^ showed a dramatic reduction in GAP-stimulated hydrolysis, with k_cat_ dropping from 2.03 s⁻¹ for RAC1^WT^ to 0.0087 s⁻¹, a 233-fold decrease (Fig. 2C, right panel). T17N and F28L mutations also reduced GAP activity but to a lesser extent (14.5-fold and 50-fold, respectively). These results underscore the critical role of GAP in the temporal regulation of RAC1 activity, with diminished p50GAP activity prolonging RAC1^P29S^’s GTP-bound state and enhancing its signaling capacity.

### RAC1^P29S^ shows a significantly stronger binding affinity to IQGAP1 compared to PAK1

The diverse signaling activities of RAC1 in human cells and cancers are primarily mediated through its interactions with downstream effectors. To evaluate the impact of the P29S mutation on effector binding under cell-free conditions, we examined its interaction with two well-characterized RAC1 effectors: the RAC1 binding domain (RBD) of the serine/threonine kinase PAK1, a key downstream kinase, and the C-terminal 794 amino acids (C794) of the scaffolding protein IQGAP1, a critical accessory protein [9, 10, 51, 52].

**Fig. 3:**
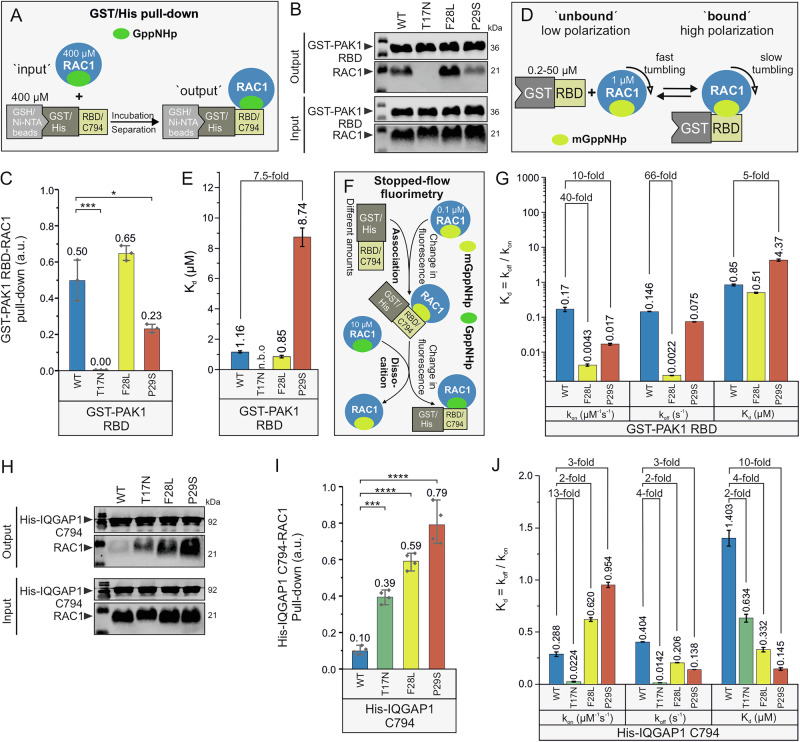
Reduced binding affinity of RAC1^P29S^ for PAK1 but increased for IQGAP1. **A** GST and His pull-down assays were performed to evaluate the binding strength of RAC1 variants to GST-PAK1 RBD and His-IQGAP1 C794, respectively. For each reaction, 50 µL of beads were incubated with 400 µM GppNHp-bound RAC1 proteins and 400 µM GST-PAK1 RBD or His-IQGAP1 C794. Input samples consisted of the protein mixtures before incubation, while output samples were the eluted fractions. **B** Western blot analysis of RAC1-PAK1 pull-down (output) was performed using anti-GST antibodies for GST-PAK1 and anti-RAC1 antibodies, with molecular weights indicated in kilodaltons (kDa). The input represents total protein mixtures before pull-down experiments. **C** Bar graphs quantify RAC1-PAK1 RBD interactions from 3 independent pull-down experiments analyzed using one-way ANOVA, with *P* values (*<0.05; **<0.01; ***<0.001; ****<0.0001) and data expressed as means ± SD. **D** The principle behind the fluorescence polarization measurements for the interaction between GST-PAK1 RBD and RAC1 proteins is illustrated. Accordingly, 1 µM mGppNHp-bound RAC1 was titrated with increasing concentrations of GST-PAK1 RBD. **E** Bar graphs from fluorescence polarization analysis represent the dissociation constants (*K*_d_) for PAK1 RBD binding to RAC1 proteins, with “n.b.o” indicating no binding observed and data expressed as means ± SD. **F** The principle behind the kinetic measurements of GST-PAK1 RBD association with and dissociation from RAC1 proteins is shown using a stopped-flow instrument. In these experiments, 0.1 µM mGppNHp-bound RAC1 was rapidly mixed with increasing concentrations of GST-PAK1 RBD to monitor association kinetics. Dissociation kinetics were measured by rapidly mixing a complex of RAC1•mGppNHp•GST-PAK1 RBD with excess GppNHp-bound RAC1. **G** Bar graphs from the stopped-flow analysis display the evaluated association rates (*k*_on_), dissociation rates (*k*_off_), and dissociation constants (*K*_d_, calculated as *k*_off_/*k*_on_) for the PAK1 RBD interaction with RAC1 proteins, with data presented as means ± SD. **H** Western blot analysis of RAC1-IQGAP1 pull-down (output) was performed using anti-His antibodies for His-IQGAP1 and anti-RAC1 antibodies, with molecular weights indicated in kilodaltons (kDa). The Input represents total protein mixtures before pull-down experiments. **I** Bar graphs quantify RAC1-IQGAP1 C794 interactions from four independent pull-down experiments analyzed using one-way ANOVA, with *P* values (*<0.05; **<0.01; ***<0.001; ****<0.0001), and data expressed as mean ± SD. **J** Bar graphs from the stopped-flow analysis depict the *k*_on_ and the *k*_off_ values for the interaction between IQGAP1 C794 and RAC1 proteins, with *K*_d_ values calculated as the ratio of *k*_off_ to *k*_on_ and all kinetic data presented as means ± SD.

The binding properties of RAC1 mutants to PAK1 RBD were assessed using a GST pull-down assay (Fig. 3A), revealing differential binding compared to RAC1^WT^: weaker binding for RAC1^P29S^, modestly stronger binding for RAC1^F28L^, and no binding for RAC1^T17N^ (Fig. 3B, C). Representative blots from the GST pull-down assay showing RAC1-PAK1 interactions are presented in Fig. 3B, with statistical analyses displayed in Fig. 3C. Fluorescence polarization further quantified these interactions, confirming no binding for RAC1^T17N^, a modest increase in affinity for RAC1^F28L^, and a 7.5-fold decrease in binding affinity for RAC1^P29S^ relative to RAC1^WT^ (Fig. 3D, E; Supplementary Fig. S7A), which displays dissociation constants (*K*_d_) derived from titrations of RAC1 mutants with GST-PAK1 RBD. The slightly enhanced affinity of RAC1^F28L^ was attributed to its slower dissociation rate. Stopped-flow fluorimetry revealed that RAC1^P29S^ and RAC1^F28L^ exhibited 10- and 40-fold slower association rates, respectively, compared to RAC1^WT^, while RAC1^F28L^ displayed a 66-fold and 34-fold slower dissociation rate compared to RAC1^WT^ and RAC1^P29S^, respectively (Fig. 3F, G; Supplementary Fig. S7B), which provides kinetic analyses of RAC1-PAK1 interactions, including association and dissociation rate constants. Overall, the binding affinity to PAK1 RBD increased slightly for RAC1^F28L^ and decreased 5-fold for RAC1^P29S^ compared to RAC1^WT^ (Fig. 3G), consistent with the results of GST pull-down and fluorescence polarization assays (Fig. 3C, E).

The interaction of IQGAP1 C794 with RAC1 variants was assessed using a His-tag pull-down assay (Fig. 3A). Binding progressively increased in the order of RAC1^WT^, RAC1^T17N^, RAC1^F28L^, and RAC1^P29S^ (Fig. 3H), which presents representative blots from the pull-down assay showing RAC1-IQGAP1 interactions, with statistical analyses displayed in Fig. 3I. This trend was confirmed by data from four independent pull-down experiments (Fig. 3I). Stopped-flow experiments further corroborated these findings, revealing a gradual increase in IQGAP1 binding affinity across the RAC1 variants in the same order (Fig. 3J; Supplementary Fig. S8), which provides kinetic analyses of RAC1-IQGAP1 interactions, including association and dissociation rate constants. Notably, RAC1^P29S^ exhibited significantly higher affinity for IQGAP1 C794 compared to PAK1 RBD, and IQGAP1 C794 bound more tightly to RAC1^T17N^ than to RAC1^WT^, providing new insights into the differential binding properties of RAC1 effectors.

### RAC1^P29S^ is found in its GTP-bound state in serum-starved HEK-293T cells

To evaluate active RAC1 levels under serum stimulation and starvation, RAC1^WT^ and mutants were overexpressed in HEK-293T cells and pulled down in their GTP-bound states using GST-PAK1 RBD, GST-IQGAP1 C794, and GST as a negative control (Supplementary Fig. S9), which provides a schematic representation of the pull-down assay used to determine the level of active, GTP-bound RAC1 from HEK-293T cell lysates.

**Fig. 4:**
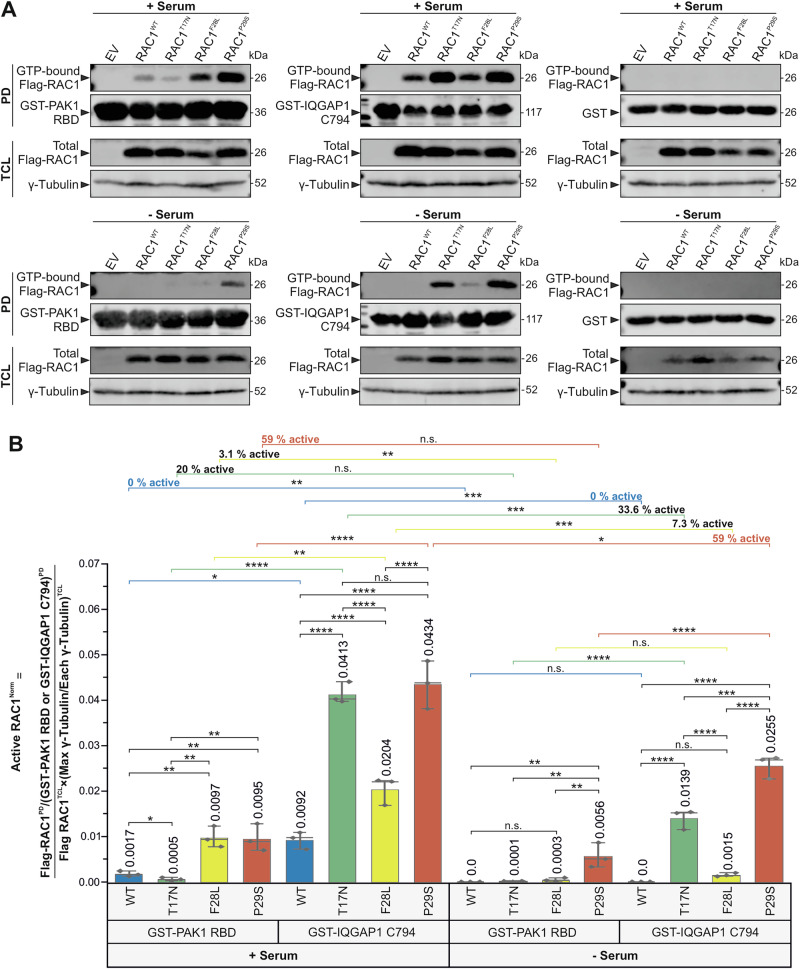
RAC1^P29S^ accumulates in its active, GTP-bound state in HEK-293T cells under serum-starved conditions. Active GTPase pull-down assays were performed to quantify GTP-bound RAC1 proteins (Supplementary Fig. S9). Lysis solutions from *E. coli* containing GST-PAK1 RBD or GST-IQGAP1 C794 were incubated with prewashed glutathione agarose beads to prepare bait-bound beads. Simultaneously, HEK-293T cells were transfected with Flag-RAC1 constructs and cultured under either serum-stimulated or serum-starved conditions for 24 h. After harvesting, the cells were lysed, and the supernatants containing GTP-bound Flag-RAC1 proteins were collected. Equal amounts of HEK cell lysates were incubated with the bait-bound beads to facilitate protein-protein interactions. After three washes to remove unbound proteins, active GTP-loaded Flag-RAC1 proteins bound to GST-PAK1 RBD or GST-IQGAP1 C794 were eluted and analyzed by SDS-PAGE and Western blotting. **A** Western blots of active GTPase pull-down assays were probed with anti-Flag, anti-GST, and anti-γ-tubulin antibodies to detect GTP-bound Flag-RAC1, GST-PAK1 RBD or GST-IQGAP1 C794, and γ-tubulin, respectively. Analyses were performed under serum-stimulated and serum-starved conditions using GST-PAK1 RBD, GST-IQGAP1 C794, and GST as negative controls. Molecular weights (in kDa) are indicated for each band corresponding to the target proteins. The pull-down (PD) lanes show the output signal representing the amount of GTP-bound Flag-RAC1 proteins captured by the bait-bound beads. GST-PAK1 RBD or GST-IQGAP1 C794 bands reflect the amount of bait protein available for RAC1 binding. Total cell lysate (TCL) lanes show Flag-RAC1 expression with γ-tubulin as a loading control. The figure consists of six Western blot panels: the first blot shows the levels of active RAC1^WT^, RAC1^T17N^, RAC1^F28L^, and RAC1^P29S^, with EV indicating the empty vector control. The upper panels show the serum-stimulated condition with GST-PAK1 RBD as bait protein (left panel), GST-IQGAP1 C794 (middle panel), and GST (right panel). The lower panels show the amount of active RAC1 after 24 h of serum starvation with GST-PAK1 RBD, GST-IQGAP1 C794, and GST from left to right. **B** Bar graphs of normalized values from three independent experiments (*n* = 3), analyzed by one-way ANOVA, were used to quantify active RAC1 proteins. *P* values are indicated as follows: *<0.05; **<0.01; ***<0.001; ****<0.0001, and n.s., not significant. Data are expressed as mean ± SD. Values for RAC1^WT^, RAC1^T17N^, RAC1^F28L^, and RAC1^P29S^ were compared and analyzed in eight different sets: Set 1 [GST-PAK1 RBD (+serum)], Set 2 [(GST-IQGAP1 C794 (+serum)], Set 3 [(GST-PAK1 RBD (+serum)) vs. (GST-IQGAP1 C794 (+serum))], Set 4 [GST-PAK1 RBD (-serum)], Set 5 [GST-IQGAP1 C794 (-serum)], Set 6 [(GST-PAK1 RBD (-serum)) vs (GST-IQGAP1 C794 (-serum))], Set 7 [(GST-PAK1 RBD (+serum) vs (-serum))], and Set 8 [(GST-IQGAP1 C794 (+serum) vs (-serum))], with the last two sets reporting the percentage of active GTP-loaded RAC1 proteins remaining from serum-stimulated to serum-starved conditions.

The results showed significantly stronger binding of GTP-bound RAC1 proteins to IQGAP1 C794 compared to PAK1 RBD under serum-stimulated conditions (Fig. 4A, upper panel). RAC1^P29S^ and RAC1^F28L^ displayed stronger binding to GST-PAK1 RBD, while RAC1^T17N^ exhibited minimal binding relative to RAC1^WT^. In contrast, all RAC1 variants showed significantly higher binding to GST-IQGAP1 C794. Under serum starvation, high levels of RAC1^P29S^•GTP were pulled down with GST-PAK1 RBD, corroborating in vitro findings and indicating temporal accumulation of RAC1^P29S^ in its GTP-bound state (Fig. 4A, lower panel). Similarly, much higher levels of RAC1^P29S^•GTP and RAC1^T17N^•GTP were pulled down with GST-IQGAP1 C794. These findings were reproduced in triplicate, with no interaction observed for GST alone.

Quantification of active, GTP-bound RAC1 levels was performed in three independent experiments for each condition (*n* = 3), where separate panels show pull-down results for GST-PAK1 RBD and GST-IQGAP1 C794 in both conditions. Results are presented as bar graphs (Fig. 4B). Under serum stimulation, in set 1 analysis, RAC1^F28L^ and RAC1^P29S^ displayed stronger binding to PAK1 RBD compared to RAC1^WT^, which showed baseline interaction, whereas RAC1^T17N^ demonstrated very weak binding, consistent with its *K*_d_ values. In set 2, RAC1^T17N^ and RAC1^P29S^ exhibited significantly stronger binding to IQGAP1 C794 compared to RAC1^WT^, which showed baseline interaction, with RAC1^F28L^ demonstrating intermediate binding. Notably, RAC1^T17N^ exhibited binding levels to IQGAP1 C794 similar to RAC1^P29S^. In set 3, all RAC1 variants bound more strongly to IQGAP1 C794 than PAK1 RBD, with RAC1^P29S^ and RAC1^T17N^ showing the highest binding levels.

Under serum starvation, set 4 showed that only RAC1^P29S^ remained active and bound to PAK1 RBD, while RAC1^WT^, RAC1^T17N^, and RAC1^F28L^ showed no significant binding. In set 5, RAC1^WT^ completely lost activity, while RAC1^T17N^ and RAC1^P29S^ remained active and strongly interacted with IQGAP1 C794, although RAC1^F28L^ activity was insufficient to achieve significance. In set 6, RAC1^WT^ lost all activity, failing to bind either PAK1 RBD or IQGAP1 C794. RAC1^T17N^ did not bind PAK1 RBD but bound strongly to IQGAP1 C794, while RAC1^F28L^ showed no significant binding to either effector. RAC1^P29S^, however, is bound more strongly to IQGAP1 C794 than to PAK1 RBD.

Sets 7 and 8 compared RAC1 activity between serum-stimulated and serum-starved conditions. RAC1^WT^ lost all activity under serum starvation, failing to bind either effector. RAC1^T17N^ retained tight binding to IQGAP1 C794 under both conditions, though binding strength decreased by 33.6% under starvation. RAC1^F28L^ lost 93–97% of its binding to PAK1 RBD and IQGAP1 C794, reflecting its fast-cycling nature. In contrast, RAC1^P29S^ retained 59% of its activity under serum starvation, binding strongly to PAK1 RBD and IQGAP1 C794, highlighting its constitutive gain-of-function properties.

### RAC1^P29S^ accumulates in its GTP-bound state in HEK-293T cells and hyperactivates cancer-related signaling pathways

**Fig. 5:**
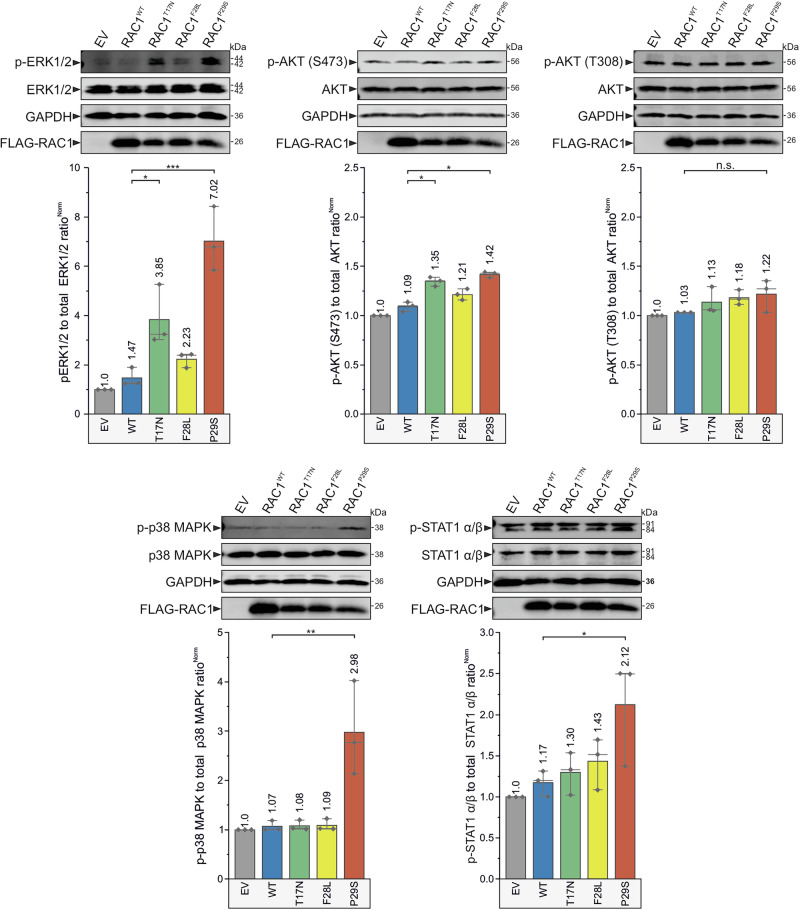
Accumulated GTP-bound RAC1^P29S^ hyperactivates various cancer signaling pathways. Immunoblot analysis was performed to evaluate the phosphorylation levels of several kinases associated with the hallmarks of oncogenic transformation. Serum-stimulated HEK-293T cells transiently overexpressing Flag-tagged RAC1^WT^, RAC1^T17N^, RAC1^F28L^, and RAC1^P29S^, along with an empty vector (EV) control, were analyzed. The phosphorylation levels of ERK1/2 and AKT (at T308 and S473) were evaluated first. Additionally, the phosphorylation of p38 MAPK was examined as a marker of cellular adaptations that enhance survival under oxidative or inflammatory stress. Finally, the phosphorylation levels of STAT1 α/β, a transcription factor downstream of p38 that may promote immune evasion and support survival under inflammatory conditions, were assessed. Phosphorylation levels were quantified by calculating the ratio of phosphorylated target proteins to total proteins (e.g., p-ERK/t-ERK) and normalizing them to GAPDH as a loading control. Flag tag detection confirmed the expression of each RAC1 variant. Representative results were obtained from three independent experiments, and statistical significance was determined using one-way ANOVA with *P* values (*P ≤ 0.05; **P ≤ 0.01; and ***P ≤ 0.001; ****P ≤ 0.0001). Data are expressed as mean ± SD.

To investigate the impact of RAC1 variants on key signaling pathways, HEK-293T cells were transiently transfected with constructs encoding Flag-tagged RAC1 variants. Western blot analysis revealed a significant increase in ERK1/2 phosphorylation (p-ERK1/2) in cells overexpressing RAC1^P29S^ (*p* < 0.001, ***) and RAC1T17N (*p* < 0.05, *) (Fig. 5). This increase was consistently observed across triplicate experiments, which present western blot analyses of phosphorylation levels of ERK1/2, AKT(S473), AKT(T308), p38 mitogen-activated protein kinase (MAPK), and STAT1 α/β in serum-stimulated HEK-293T cells overexpressing RAC1 variants. The observed ERK hyperactivation aligns with its established role in promoting tumor growth and proliferation. RAC1^P29S^ also significantly elevated p38 MAPK phosphorylation (*p* < 0.01, **) (Fig. 5). Phosphorylation of protein kinase (AKT) at serine 473 (S473), a target of mTORC2, and STAT1 α/β phosphorylation were statistically significant (*p* < 0.05, *), but less pronounced compared to ERK and p38 MAPK. AKT phosphorylation at threonine 308 (T308), a PDK1 target, remained non-significant (n.s.).

### Constitutive RAC1^P29S^ activation in IGR1 melanoma cells is significantly reduced by DOCK2 inhibition

**Fig. 6:**
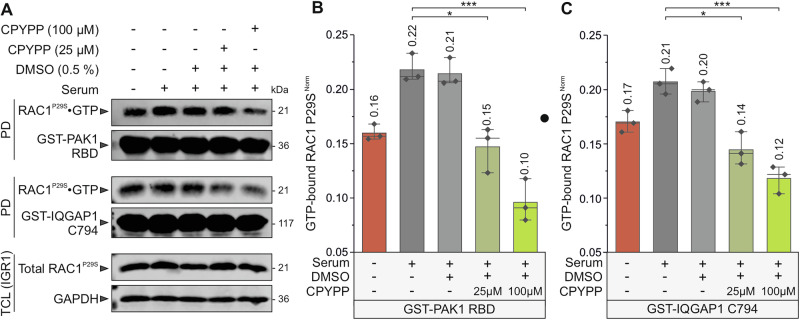
Constitutive RAC1^P29S^ activation in IGR1 melanoma cells is significantly reduced by DOCK2 inhibition. Active GTPase pull-down assays were performed to quantify the amount of GTP-bound RAC1^P29S^ protein (see Supplementary Fig. S9). Lysates from *E. coli* expressing GST-PAK1 RBD or GST-IQGAP1 C794 were incubated with prewashed glutathione agarose beads to generate bait-bound beads. IGR1 melanoma cells that expressing the RAC1^P29S^ mutant endogenously were cultured under either serum-starved or serum-stimulated conditions. The cells were then treated with 0.5% DMSO, and with 25 or 100 µM of the DOCK2 inhibitor CPYPP for 3 h. After treatment, cells were lysed, and GTP-bound RAC1^P29S^-containing supernatants were collected. Equal amounts of IGR1 lysates were incubated with the bait-bound beads to capture the active RAC1^P29S^ protein. After washing to remove unbound proteins, the bound GTP-loaded RAC1^P29S^ was eluted and analyzed by SDS-PAGE and Western blotting. **A** Western blots of the pull-down samples were probed with anti-RAC1, anti-GST, and anti-GAPDH antibodies to detect GTP-bound RAC1^P29S^, GST-PAK1 RBD or GST-IQGAP1 C794, and GAPDH, respectively. Molecular weights (in kDa) are indicated for each band. The pull-down (PD) lanes represent the amount of active RAC1^P29S^ captured by the bait-bound beads. The GST-PAK1 RBD and GST-IQGAP1 C794 bands reflect the levels of the bait protein. The total cell lysate (TCL) lanes demonstrate endogenous RAC1^P29S^ expression, with GAPDH serving as a loading control. **B**, **C** Bar graphs show normalized quantification (normalized to GAPDH, endogenous total RAC1^P29S^ levels, and bait protein amounts) from three independent experiments (*n* = 3), analyzed by one-way ANOVA. *P* values are indicated as follows: *P ≤ 0.05; **P ≤ 0.01; and ***P ≤ 0.001.

To investigate the functional impact of the P29S mutation on RAC1 activity in melanoma cells and to validate our biochemical findings in HEK-293T cells, we used IGR1 human melanoma cells, which express the RAC1^P29S^ mutant endogenously. First, we examined whether RAC1^P29S^ accumulates in its active, GTP-bound state under serum-starved conditions, where GEF activity is minimal and RAC1 is expected to largely be GDP-bound. Second, we examined the effect of DOCK2 inhibition on RAC1^P29S^ activation in serum-stimulated IGR1 cells treated with CPYPP, a compound that binds to the DHR2 domain of DOCK2 and inhibits its GEF activity. Under serum-starved conditions, we observed a strong accumulation of active RAC1^P29S^•GTP, as detected by both GST–PAK1 RBD and GST–IQGAP1 C794. This result confirms our previous findings in HEK-293T cells. These data support the notion of impaired GAP-mediated GTP hydrolysis, as demonstrated previously in both the GAP assay and GTPase pull-down assays. Furthermore, CPYPP treatment reduced RAC1^P29S^ activation in a dose-dependent manner, with significantly reductions at both concentrations: 25 µM (*P* ≤ 0.05, *) and 100 µM (*P* ≤ 0.001, ***). Treatment with 0.5% DMSO alone had no detectable effect. These results confirm the pivotal role of DOCK2 in regulating RAC1^P29S^ activation. This role was demonstrated in both the in vitro GEF assay and in cell-based experiments using IGR1 melanoma cells. The results also suggest that targeting DOCK2 is an effective inhibitory strategy for counteracting RAC1^P29S^-driven melanoma cell invasion (Fig. 6).

## Discussion

**Fig. 7:**
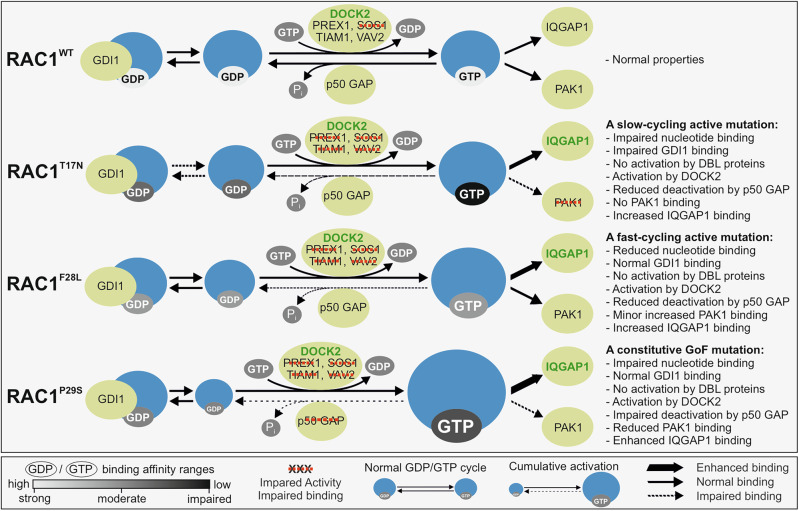
This schematic summarizes the findings of this study, which focuses on the biochemical characterization of RAC1^T17N^, RAC1^F28L^, and RAC1^P29S^ mutants in comparison to RAC1^WT^. The middle section of the figure includes key guides illustrating the strength of GDP/GTP binding, impaired versus enhanced activity or binding to regulators and effectors, and the distinction between the normal GDP/GTP cycle and cumulative activation. Compared to RAC1^WT^, the RAC1^P29S^ mutant significantly impairs nucleotide binding and exhibits a rapid intrinsic nucleotide exchange rate, while the RAC1^T17N^ mutant shows the most impaired nucleotide binding overall. The P29S mutation has a minimal effect on RAC1-GDI1 interaction, whereas the T17N mutation severely impairs GDI1 activity. The P29S mutation is predominantly activated by DOCK2 rather than DBL family GEFs, with GEF-mediated nucleotide exchange being impaired. A key finding of this study is that the P29S mutation significantly impairs GAP-stimulated GTP hydrolysis of RAC1, providing a temporal mechanism for the accumulation of RAC1^P29S^ in its GTP-bound active form and driving its hyperactivation. While the T17N variant shows no binding affinity for PAK1, the P29S mutation demonstrates a dual effect in vitro: reduced binding affinity for PAK1 but enhanced affinity for IQGAP1. This highlights the pivotal role of accessory proteins, particularly IQGAP1, in driving RAC1^P29S^-mediated downstream activation. The rightmost section of the figure provides a detailed summary of the biochemical properties of the RAC1 proteins analyzed in this study.

This study provides a comprehensive biochemical characterization of RAC1^P29S^ in comparison to RAC1^WT^, RAC1^T17N^, and RAC1^F28L^ (Fig. 7). Our findings reveal that (i) RAC1^P29S^ exhibits impaired nucleotide binding and accelerated intrinsic nucleotide exchange; (ii) its activation is primarily mediated by DOCK2 rather than DBL family GEFs; (iii) GAP-stimulated GTP hydrolysis is severely impaired, enabling persistent accumulation of RAC1^P29S^ in its active, GTP-bound state; (iv) RAC1^P29S^ exhibits stronger binding to IQGAP1 than to PAK1, highlighting IQGAP1 as a key spatial modulator of its downstream signaling; and (v) the accumulation of GTP-bound RAC1^P29S^ leads to hyperactivation of key cancer-associated signaling pathways, including ERK1/2, p38 MAPK. Importantly, we validated these biochemical features in IGR1 human melanoma cells, which express RAC1^P29S^ endogenously. Active GTP-bound RAC1^P29S^ accumulated under serum-starved conditions, confirming the presence of impaired GAP regulation in a context relevant to cancer. Furthermore, pharmacological inhibition of DOCK2 with CPYPP significantly reduced RAC1^P29S^ activation, establishing DOCK2 as a pivotal upstream activator in melanoma. Taken together, these results classify RAC1^P29S^ as a constitutively active, gain-of-function mutant and an oncogene (Box 1) that transduces upstream signals to effectors such as IQGAP1, thereby promoting melanoma progression through enhanced proliferation, invasion, and epithelial-to-mesenchymal transition [53, 54].

Our biochemical data confirm that the P29S mutation increases the intrinsic nucleotide exchange rate, consistent with previous reports [36, 37]. The slower GDP/GTP association rate results in a reduced nucleotide binding affinity despite accelerated exchange. Shimada et al. demonstrated that RAC1P29S enhances GDP dissociation, favoring a GTP-bound state that drives oncogenic activity [55]. Similarly, Gursoy et al. used molecular dynamics to show that this mutation increases switch I flexibility, facilitating rapid GDP/GTP exchange [20]. Our findings suggest that the elevated exchange rate and activation of RAC1P29S arise from impaired nucleotide-binding affinity due to conformational changes induced by the P29S substitution. However, the intrinsic exchange rate of RAC1^P29S^ remains insufficient for many cellular processes, emphasizing the importance of GEF-mediated exchange in its activation in cancer cells.

RHO-specific GDIs regulate RHO GTPase dynamics by extracting them from membranes, maintaining their inactive state, and preventing degradation through specific interactions [1]. Despite progress in understanding GDI-mediated shuttling, some mechanisms remain unclear. We previously showed that GDI1 binds RAC1 regardless of its prenylation state [8]. Our data suggest that RAC1^T17N^ has impaired GDI1 activity, with decreased binding affinity, which may suggest persistent plasma membrane association. In contrast, RAC1^P29S^ shows only a slight reduction in GDI1 affinity, indicating that GDI1 can still modulate its localization and translocation.

RAC1^P29S^, like most oncogenes, requires repeated activation by RAC1-specific GEFs. Our data demonstrate that RAC1 mutants exhibit minimal activation by DBL family GEFs, such as TIAM1, PREX1, and VAV2, while DOCK2 significantly enhances the exchange rate for all RAC1 variants, including P29S. This observation aligns with the distinct mechanistic roles of the P-loop and switch I in RAC1, particularly in the functions of DBL and DOCK GEF families [7, 41, 56, 57]. However, further analysis is needed to fully understand RAC1^P29S^ activation in cancer cells. Uruno et al. showed that DOCK1 inhibition suppresses cancer cell invasion and macropinocytosis induced by RAC1^P29S^ in melanoma and breast cancer cells [57]. Notably, DOCK2 is a potent RAC1 activator in cancers, including melanoma and chronic lymphocytic leukemia [58–60], and regulates critical processes such as lymphocyte migration, T-cell differentiation, cell-cell adhesion, and bone marrow homing of immune cells [61]. Although slight increases in TIAM1 activity were observed in our study, the TIAM1-RAC1 axis cannot be entirely excluded from RAC1^P29S^ activation in cancers, including melanoma [62].

Consistent with its traditionally assumed dominant negative behavior, RAC1^T17N^ did not show increased GEF-mediated nucleotide exchange via DBL proteins. However, our findings reveal additional regulatory and functional properties that challenge this simplistic interpretation. In HEK-293T cells, RAC1^T17N^ overexpression significantly increased ERK phosphorylation, albeit less than RAC1^P29S^. This effect was further enhanced under serum-stimulated conditions. These observations suggest that RAC1^T17N^ may function as a slow-cycling, partially active variant rather than a strictly dominant-negative mutant (Fig. 7). Consistent with this idea, we detected measurable levels of GTP-bound RAC1^T17N^ in cell lysates, particularly under serum stimulation. This is likely driven by DOCK2-mediated nucleotide exchange (Fig. 2B). Furthermore, RAC1^T17N^ interacted with IQGAP1, a scaffolding protein that promotes MAPK pathway activation. This interaction was observed both in vitro using purified GppNHp-bound active RAC1^T17N^ (Fig. 3H–J) and in cell-based assays (Fig. 4) and may account for the elevated ERK phosphorylation (Fig. 5). It indicates RAC1^T17N^ exhibits context-dependent functional signaling activity. Cool et al. demonstrated that HRAS^D119N^ exhibits dose-dependent dominant-negative and constitutively active effects by reducing nucleotide affinity, sequestering GEFs, binding GTP independently of GEFs, and activating downstream pathways at high concentrations [63]. A similar dual behavior may apply to RAC1^T17N^, depending on cellular conditions and expression levels. Similarly, RAC1^P29S^ signaling may partially result from overexpression. RAC1^F28L^ has a GEF activity profile similar to that of RAC1^P29S^. This suggests that both mutations may alter the RAC1 GEF-binding interface in a similar manner. Further structural investigation of this possibility is warranted.

Among the analyzed DBL proteins, SOS1 showed no activity. Other DBL proteins, such as ABR, α-PIX, β-PIX, BCR, FGD4, and FGD6, contain pseudo-DH domains with functions yet to be determined [3]. These domains, defined as globular structures performing specific roles like binding or catalysis independent of full-length protein context, may require posttranslational modifications [64–66] or interactions with specific binding partners [67] to become active.

RAC1 signaling is terminated by GTP hydrolysis to GDP, deactivating the protein [5]. The intrinsic GTP hydrolysis rate of RAC1^WT^ and its mutants is slow (~9000 s), necessitating GAPs to catalyze hydrolysis and reduce deactivation time to just a second [48]. Our findings confirm that RAC1^P29S^ retains a similar intrinsic hydrolysis rate to RAC1^WT^ [36, 37]. However, this study reveals for the first time that the P29S mutation severely impairs GAP-mediated hydrolysis, with p50GAP activity reducing the inactivation time of RAC1^P29S^ to ~1000 s, a 233-fold decrease compared to RAC1^WT^.

Previous studies have classified RAC1^P29S^ as a spontaneously activating, self-activating, fast-cycling mutant [19, 36, 37] or an oncogenic driver [68] due to its rapid nucleotide exchange that maintains RAC1 in an active state (Box 1). Our findings align with the latter, highlighting the critical role of p50GAP in regulating RAC1^P29S^ activity. The severe impairment of GAP-stimulated GTP hydrolysis supports its classification as a constitutive gain-of-function mutant and an oncogene, driven by defective GAP-mediated deactivation rather than just increased nucleotide exchange. This disruption in temporal regulation leads to the accumulation of active RAC1^P29S^•GTP, as confirmed by its persistence in the GTP-bound state under serum-starved conditions, where most GTPases are typically inactive due to GAP sensitivity and lack of upstream GEF activation. As supported by prior studies, the sustained activation of RAC1^P29S^ likely drives cancer-related processes, including proliferation, survival, invasion, metastasis, and therapy resistance [19, 23–25, 27, 36, 69–71].

The diverse signaling activities of RAC1 are mediated through interactions with specific effectors, which require RAC1 to adopt distinct conformations to function [1]. RAC1 effectors include kinases such as PAK1/2/3, MLK1, PI4P5Ks, and accessory proteins like IQGAP1/2, IRSP53, AJUBA, p67phox, and CYFIP1/2 [1]. This study examined the binding properties of PAK1, a major kinase, and IQGAP1, a critical scaffolding protein. IQGAP1 is involved in cytoskeletal reorganization processes, including polarity, adhesion, and migration [72, 73], and links RAC1 to the actin cytoskeleton via filamentous actin binding [74]. Previous studies showed IQGAP1 interacts with RAC1 and CDC42 via switch regions and effector binding sites, with slight differences in mechanisms [2, 43, 51, 52]. Malliri et al. demonstrated that IQGAP1 exhibits increased RAC1 binding specifically upon TIAM1 expression but not other DBL GEFs, including PREX1 [75].

Our findings reveal that RAC1^P29S^ interacts significantly more strongly with IQGAP1 than with PAK1, exhibiting a 30-fold higher binding affinity as measured by stopped-flow fluorimetry. This enhanced interaction was corroborated by a statistically significant increase in RAC1^P29S^•GTP binding to IQGAP1 under both serum-stimulated and serum-starved conditions. In contrast, the stronger binding of RAC1^P29S^ to GST-PAK1 RBD observed in human cell lysates, compared to in vitro pull-down assays using purified proteins, may be attributed to the presence of accessory proteins [76], modulators, other cellular components and/or compensatory pathways [77] that facilitate protein complex formation and RAC1-effector interactions in the native cellular environment. These findings suggest that IQGAP1 is a key effector downstream of RAC1^P29S^, acting as an activated scaffolding protein to modulate pathways such as RAF/MEK/ERK [78–80]. This underscores the pivotal role of scaffolding proteins, particularly IQGAP1, as spatial modulators facilitating RAC1^P29S^-driven signaling and its downstream effects.

Although RAC1^P29S^ exhibits stronger binding to IQGAP1 than to PAK1 in vitro, its elevated GTP-bound state in cell lysates, as demonstrated in the GST–PAK1 RBD pull-down (Fig. 4A), indicates its constitutive activation rather than an enhanced direct affinity for PAK1. This distinction stems from the different experimental contexts: in vitro binding assays (Fig. 3) measure intrinsic interaction strength under defined nucleotide states, while pull-downs from cell lysates capture the abundance of active RAC1^P29S^ in a native environment. Elevated levels of GTP-bound RAC1^P29S^ can result in increased downstream signaling, including PAK1 activation, as previously demonstrated by Downward and colleagues. They reported elevated phospho-PAK1/2 levels and AKT pathway activation in RAC1^P29S^-expressing melanoma cells [25]. These findings support the functional relevance of our pull-down results and reinforce the interpretation that RAC1^P29S^ acts as a constitutively active mutant capable of engaging multiple effectors in a context-dependent manner.

Hyperactivation of signaling pathways downstream of RAC1^P29S^ highlights its oncogenic potential. Accumulated GTP-bound RAC1^P29S^ robustly enhances ERK1/2 and p38 MAPK phosphorylation, suggesting these pathways play significant roles in RAC1^P29S^-driven oncogenic transformation. ERK hyperactivation, a hallmark of uncontrolled tumor growth and proliferation, promotes unregulated cell cycle progression. Concurrently, p38 MAPK hyperactivation supports cellular adaptation to oxidative and inflammatory stress, contributing to tumor progression, invasion, and therapeutic resistance. These findings highlight ERK and p38 MAPK as important mediators of RAC1^P29S^-driven oncogenic signaling, while acknowledging additional pathways may also contribute. Phosphorylation of AKTS473, mediated by mTORC2, and STAT1 α/β, while statistically significant, was less pronounced and may represent secondary or context-specific effects. Selective AKTS473 activation could support cancer cell survival and metabolic adaptation, while STAT1 hyperactivation might facilitate immune evasion and survival under inflammatory conditions. This study focused on these pathways to illustrate GTP-bound RAC1^P29S^ hyperactivation and validate cell-free data highlighting its constitutive activation. However, many other signaling events remain unexplored, underscoring the need for future studies to fully elucidate RAC1^P29S^-driven cancer mechanisms.

To validate our biochemical data and expand its applicability to melanoma, we examined RAC1^P29S^ activity in IGR1 human melanoma cells, which harbor both the *RAC1 P29S* and *BRAF V600K* mutations endogenously. Consistent with our HEK-293T experiments, we found that RAC1^P29S^ remains constitutively GTP-bound even under serum-starved conditions. This finding reinforces the idea that impaired GAP-mediated hydrolysis is the cause of its sustained activation. Furthermore, DOCK2 inhibition using CPYPP significantly reduced RAC1^P29S^ activity in a dose-dependent manner, which supports the central role of DOCK2 as the primary GEF responsible for RAC1^P29S^ activation in melanoma cells. Previous studies have characterized CPYPP as a small-molecule inhibitor that binds the DHR2 domain of DOCK2, reversibly blocking its catalytic activity and downstream RAC-mediated signaling responses [81]. These findings highlight DOCK2 as a potential therapeutic target in RAC1-mutant melanoma. IGR1 cells, which co-express RAC1^P29S^ and BRAF ^V600K^, have been shown to exhibit reduced sensitivity to BRAF inhibitors such as vemurafenib and dabrafenib due to RAC1^P29S^-driven resistance mechanisms [24]. Enforced expression of RAC1^P29S^ increased cell survival, stimulates tumor growth, and inhibits apoptosis when RAF inhibitors are present. Conversely, knockdown of RAC1^P29S^ restores drug sensitivity [24]. Mechanistically, RAC1^P29S^ has been shown to activate the PAK and AKT pathways and to drive a mesenchymal phenotypic switch via the SRF/MRTF transcriptional axis and promote melanoma progression and therapeutic resistance [25]. Additionally, RAC1^P29S^ promotes lamellipodia formation and cytoskeletal remodeling via Arp2/3-mediated actin polymerization. This contributes to enhanced proliferation and invasion even in growth-suppressive environments [27]. RAC1^P29S^-induced matrix invasion and macropinocytosis have also been shown to depend on DOCK1 activity and can be blocked by selective DOCK1 inhibition [57]. Together, our findings provide direct evidence that RAC1^P29S^ is constitutively active in melanoma cells and that DOCK2-mediated activation contributes to its pathological function. These results support the concept of targeting DOCK family GEFs, particularly DOCK2, as part of a co-inhibition strategy to suppress RAC1^P29S^-driven signaling, reducing melanoma progression, invasion, and resistance to BRAF inhibitors.

## Conclusion

This study highlights the oncogenic potential of the RAC1^P29S^ mutation by demonstrating its accumulation in the GTP-bound state, which results in the hyperactivation of downstream signaling pathways. The P29S mutation significantly impairs nucleotide binding and accelerates intrinsic nucleotide exchange. RAC1^P29S^ is primarily activated by DOCK2, rather than by DBL family GEFs, and exhibits severely impaired p50GAP-mediated GTP hydrolysis. This defective inactivation mechanism allows for the accumulation of active RAC1^P29S^•GTP over time and promotes the hyperactivation of cancer-associated pathways, including ERK and p38 MAPK. Our findings also show that RAC1^P29S^ interacts preferentially with the scaffolding protein IQGAP1, which likely serves as a key spatial modulator of its downstream signaling. Most importantly, we demonstrate that RAC1^P29S^ remains constitutively active in IGR1 human melanoma cells even under serum-starved conditions and that its activation can be significantly reduced by the pharmacological inhibition of DOCK2 with CPYPP. These cell-based data validate our biochemical results and underscore the critical role of DOCK2 in maintaining RAC1^P29S^-driven oncogenic signaling in melanoma cells. Taken together, these results establish RAC1^P29S^ as a constitutively active driver of tumorigenesis and support the concept of targeting both its upstream regulators (DOCK2 and p50GAP) and downstream effectors (IQGAP1) as part of a co-inhibition strategy to suppress RAC1^P29S^-mediated melanoma progression, invasion, and resistance to targeted therapies.

## Supplementary information

Supplementary Information

Original data files

## Data Availability

All referenced data sources are openly accessible and are cited appropriately within the manuscript. Please contact the corresponding author if you require any additional information or clarification.

## References

[1] Mosaddeghzadeh N, Ahmadian MR. The RHO family GTPases: mechanisms of regulation and signaling. Cells. 2021;10:1831. 34359999 10.3390/cells10071831 PMC8305018

[2] Dvorsky R, Ahmadian MR. Always look on the bright site of Rho: structural implications for a conserved intermolecular interface. EMBO Rep. 2004;5:1130–6. 15577926 10.1038/sj.embor.7400293 PMC1299188

[3] Jaiswal M, Dvorsky R, Ahmadian MR. Deciphering the molecular and functional basis of Dbl family proteins: a novel systematic approach toward classification of selective activation of the Rho family proteins. J Biol Chem. 2013;288:4486–500. 23255595 10.1074/jbc.M112.429746 PMC3567697

[4] Koubek EJ, Santy LC. Actin up: an overview of the Rac GEF Dock1/Dock180 and its role in cytoskeleton rearrangement. Cells. 2022;11:3565. 36428994 10.3390/cells11223565 PMC9688060

[5] Amin E, Jaiswal M, Derewenda U, Reis K, Nouri K, Koessmeier KT, et al. Deciphering the molecular and functional basis of RHOGAP family proteins: a systematic approach toward selective inactivation of RHO family proteins. J Biol Chem. 2016;291:20353–71. 27481945 10.1074/jbc.M116.736967 PMC5034035

[6] Maldonado MDM, Medina JI, Velazquez L, Dharmawardhane S. Targeting Rac and Cdc42 GEFs in metastatic cancer. Front Cell Dev Biol. 2020;8:201. 32322580 10.3389/fcell.2020.00201 PMC7156542

[7] Boland A, Côté JF, Barford D. Structural biology of DOCK-family guanine nucleotide exchange factors. FEBS Lett. 2023;597:794–810. 36271211 10.1002/1873-3468.14523 PMC10152721

[8] Mosaddeghzadeh N, Kazemein Jasemi NS, Majolée J, Zhang S-C, Hordijk PL, Dvorsky R, et al. Electrostatic forces mediate the specificity of RHO GTPase-GDI interactions. Int J Mol Sci. 2021;22:12493. 34830380 10.3390/ijms222212493 PMC8622166

[9] Fiegen D, Haeusler L-C, Blumenstein L, Herbrand U, Dvorsky R, Vetter IR, et al. Alternative splicing of Rac1 generates Rac1b, a self-activating GTPase. J Biol Chem. 2004;279:4743–9. 14625275 10.1074/jbc.M310281200

[10] Haeusler LC, Blumenstein L, Stege P, Dvorsky R, Ahmadian MR. Comparative functional analysis of the Rac GTPases. FEBS Lett. 2003;555:556–60. 14675773 10.1016/s0014-5793(03)01351-6

[11] Bailly C, Beignet J, Loirand G, Sauzeau V. Rac1 as a therapeutic anticancer target: Promises and limitations. Biochem Pharm. 2022;203:115180. 35853497 10.1016/j.bcp.2022.115180

[12] Porter AP, Papaioannou A, Malliri A. Deregulation of Rho GTPases in cancer. Small GTPases. 2016;7:123–38. 27104658 10.1080/21541248.2016.1173767 PMC5003542

[13] Alan JK, Lundquist EA. Mutationally activated Rho GTPases in cancer. Small GTPases. 2013;4:159–63. 24088985 10.4161/sgtp.26530 PMC3976972

[14] Hodge RG, Schaefer A, Howard SV, Der CJ. RAS and RHO family GTPase mutations in cancer: twin sons of different mothers?. Crit Rev Biochem Mol Biol. 2020;55:386–407. 32838579 10.1080/10409238.2020.1810622

[15] Priolo M, Zara E, Radio FC, Ciolfi A, Spadaro F, Bellacchio E, et al. Clinical profiling of MRD48 and functional characterization of two novel pathogenic RAC1 variants. Eur J Hum Genet. 2023;31:805–14. 37059841 10.1038/s41431-023-01351-7 PMC10326044

[16] Kotelevets L, Chastre E. Rac1 signaling: from intestinal homeostasis to colorectal cancer metastasis. Cancers. 2020;12:665. 32178475 10.3390/cancers12030665 PMC7140047

[17] Vu HL, Rosenbaum S, Purwin TJ, Davies MA, Aplin AE. RAC 1 P29S regulates PD-L1 expression in melanoma. Pigment Cell Melanoma Res. 2015;28:590–8. 26176707 10.1111/pcmr.12392 PMC4675336

[18] Foth M, Parkman G, Battistone B, McMahon M. RAC1mutation is not a predictive biomarker for PI3’-kinase-β-selective pathway-targeted therapy. Pigment Cell Melanoma Res. 2020;33:719–30. 32406574 10.1111/pcmr.12889 PMC13235968

[19] Krauthammer M, Kong Y, Ha BH, Evans P, Bacchiocchi A, McCusker JP, et al. Exome sequencing identifies recurrent somatic RAC1 mutations in melanoma. Nat Genet. 2012;44:1006–14. 22842228 10.1038/ng.2359 PMC3432702

[20] Senyuz S, Jang H, Nussinov R, Keskin O, Gursoy A. Mechanistic differences of activation of Rac1P29S and Rac1A159V. J Phys Chem B. 2021;125:3790–802. 33848152 10.1021/acs.jpcb.1c00883 PMC8154616

[21] Revach O-Y, Winograd-Katz SE, Samuels Y, Geiger B. The involvement of mutant Rac1 in the formation of invadopodia in cultured melanoma cells. Exp cell Res. 2016;343:82–8. 26873115 10.1016/j.yexcr.2016.02.003 PMC4954600

[22] King SJ, Asokan SB, Haynes EM, Zimmerman SP, Rotty JD, Alb JrJG, et al. Lamellipodia are crucial for haptotactic sensing and response. J Cell Sci. 2016;129:2329–42. 27173494 10.1242/jcs.184507 PMC4920251

[23] Zhu EY, Schillo JL, Murray SD, Riordan JD, Dupuy AJ. Understanding cancer drug resistance with Sleeping Beauty functional genomic screens: application to MAPK inhibition in cutaneous melanoma. iScience. 2023;26:107805. 10.1016/j.isci.2023.107805 PMC10582486 37860756

[24] Watson IR, Li L, Cabeceiras PK, Mahdavi M, Gutschner T, Genovese G, et al. The RAC1 P29S hotspot mutation in melanoma confers resistance to pharmacological inhibition of RAF. Cancer Res. 2014;74:4845–52. 25056119 10.1158/0008-5472.CAN-14-1232-T PMC4167745

[25] Lionarons DA, Hancock DC, Rana S, East P, Moore C, Murillo MM, et al. RAC1P29S induces a mesenchymal phenotypic switch via serum response factor to promote melanoma development and therapy resistance. Cancer cell. 2019;36:68–83.e9. 31257073 10.1016/j.ccell.2019.05.015 PMC6617390

[26] Cannon AC, Budagyan K, Uribe-Alvarez C, Kurimchak AM, Araiza-Olivera D, Cai KQ, et al. Unique vulnerability of RAC1-mutant melanoma to combined inhibition of CDK9 and immune checkpoints. Oncogene. 2024;43:729–43. 10.1038/s41388-024-02947-z PMC11157427 38243078

[27] Mohan AS, Dean KM, Isogai T, Kasitinon SY, Murali VS, Roudot P, et al. Enhanced dendritic actin network formation in extended lamellipodia drives proliferation in growth-challenged Rac1P29S melanoma cells. Dev cell. 2019;49:444–60.e9. 31063759 10.1016/j.devcel.2019.04.007 PMC6760970

[28] Gadal S, Boyer JA, Roy SF, Outmezguine NA, Sharma M, Li H, et al. Tumorigenesis driven by the BRAF V600E oncoprotein requires secondary mutations that overcome its feedback inhibition of migration and invasion. Cancer Res. 2025;85:1611–27. 10.1158/0008-5472.CAN-24-2220 PMC12046322 39992718

[29] Immisch L, Papafotiou G, Gallarín Delgado N, Scheuplein V, Paschen A, Blankenstein T, et al. Targeting the recurrent Rac1P29S neoepitope in melanoma with heterologous high-affinity T cell receptors. Front Immunol. 2023;14:1119498. 36875127 10.3389/fimmu.2023.1119498 PMC9978334

[30] Maldonado MDM, Dharmawardhane S. Targeting rac and Cdc42 GTPases in cancer. Cancer Res. 2018;78:3101–11. 29858187 10.1158/0008-5472.CAN-18-0619 PMC6004249

[31] Liang J, Oyang L, Rao S, Han Y, Luo X, Yi P, et al. Rac1, a potential target for tumor therapy. Front Oncol. 2021;11:674426. 34079763 10.3389/fonc.2021.674426 PMC8165220

[32] Ma N, Xu E, Luo Q, Song G. Rac1: A regulator of cell migration and a potential target for cancer therapy. Molecules. 2023;28:2976. 37049739 10.3390/molecules28072976 PMC10096471

[33] Colón-Bolea P, García-Gómez R, Casar B. RAC1 activation as a potential therapeutic option in metastatic cutaneous melanoma. Biomolecules. 2021;11:1554. 34827551 10.3390/biom11111554 PMC8615836

[34] Sauzeau V, Beignet J, Vergoten G, Bailly C. Overexpressed or hyperactivated Rac1 as a target to treat hepatocellular carcinoma. Pharm Res. 2022;179:106220. 10.1016/j.phrs.2022.106220 35405309

[35] De P, Rozeboom BJ, Aske JC, Dey N. Active RAC1 promotes tumorigenic phenotypes and therapy resistance in solid tumors. Cancers. 2020;12:1541. 32545340 10.3390/cancers12061541 PMC7352592

[36] Davis MJ, Ha BH, Holman EC, Halaban R, Schlessinger J, Boggon TJ. RAC1P29S is a spontaneously activating cancer-associated GTPase. Proc Natl Acad Sci USA. 2013;110:912–7. 23284172 10.1073/pnas.1220895110 PMC3549122

[37] Kawazu M, Ueno T, Kontani K, Ogita Y, Ando M, Fukumura K, et al. Transforming mutations of RAC guanosine triphosphatases in human cancers. Proc Natl Acad Sci USA. 2013;110:3029–34. 23382236 10.1073/pnas.1216141110 PMC3581941

[38] Li A, Machesky LM. Rac1 cycling fast in melanoma with P29 S. Pigment Cell Melanoma Res. 2013;26:289–290. 10.1111/pcmr.12074 23530970

[39] Acuner SE, Sumbul F, Torun H, Haliloglu T. Oncogenic mutations on Rac1 affect global intrinsic dynamics underlying GTP and PAK1 binding. Biophys J. 2021;120:866–76. 33515600 10.1016/j.bpj.2021.01.016 PMC8008323

[40] Lin R, Bagrodia S, Cerione R, Manor D. A novel Cdc42Hs mutant induces cellular transformation. Curr Biol. 1997;7:794–7. 9368762 10.1016/s0960-9822(06)00338-1

[41] Rajendran V, Gopalakrishnan C, Purohit R. Impact of point mutation P29S in RAC1 on tumorigenesis. Tumor Biol. 2016;37:15293–304. 10.1007/s13277-016-5329-y 27699663

[42] Parise A, Magistrato A. Assessing the mechanism of fast-cycling cancer-associated mutations of Rac1 small Rho GTPase. Protein Sci. 2024;33:e4939. 38501467 10.1002/pro.4939 PMC10949326

[43] Nouri K, Fansa EK, Amin E, Dvorsky R, Gremer L, Willbold D, et al. IQGAP1 interaction with RHO family proteins revisited: kinetic and equilibrium evidence for multiple distinct binding sites. J Biol Chem. 2016;291:26364–76. 27815503 10.1074/jbc.M116.752121 PMC5159498

[44] Hemsath L, Ahmadian MR. Fluorescence approaches for monitoring interactions of Rho GTPases with nucleotides, regulators, and effectors. Methods. 2005;37:173–82. 16289968 10.1016/j.ymeth.2005.05.014

[45] Eberth A, Ahmadian MR. In vitro GEF and GAP assays. Curr Protoc Cell Biol. 2009;43:14.9. 1–.9. 25. 10.1002/0471143030.cb1409s43 19499504

[46] Haeusler LC, Hemsath L, Fiegen D, Blumenstein L, Herbrand U, Stege P, et al. Purification and biochemical properties of Rac1, 2, 3 and the splice variant Rac1b. Methods Enzymol. 2006;406:1–11. 16472645 10.1016/S0076-6879(06)06001-0

[47] Mohr I, Mirzaiebadizi A, Sanyal SK, Chuenban P, Ahmadian MR, Ivanov R, et al. Characterization of the small Arabidopsis thaliana GTPase and ADP-ribosylation factor-like 2 protein TITAN 5. J Cell Sci. 2024;137:jcs262315. 10.1242/jcs.262315 PMC11361645 39056156

[48] Eberth A, Dvorsky R, Becker CF, Beste A, Goody RS, Ahmadian MR. Monitoring the real-time kinetics of the hydrolysis reaction of guanine nucleotide-binding proteins. Biol Chem. 2005;386:1105–14. 10.1515/BC.2005.127 16307476

[49] Herbrand U, Reza Ahmadian M. p190-RhoGAP as an integral component of the Tiam1/Rac1-induced downregulation of Rho. Biol Chem. 2006;387:331–7. 10.1515/BC.2006.041 16542153

[50] Kulkarni K, Yang J, Zhang Z, Barford D. Multiple factors confer specific Cdc42 and Rac protein activation by dedicator of cytokinesis (DOCK) nucleotide exchange factors. J Biol Chem. 2011;286:25341–51. 21613211 10.1074/jbc.M111.236455 PMC3137105

[51] Nouri K, Timson DJ, Ahmadian MR. New model for the interaction of IQGAP1 with CDC42 and RAC1. Small GTPases. 2020;11:16–22. 28622072 10.1080/21541248.2017.1321169 PMC6959280

[52] Mosaddeghzadeh N, Nouri K, Krumbach OH, Amin E, Dvorsky R, Ahmadian MR. Selectivity determinants of RHO GTPase binding to IQGAPs. Int J Mol Sci. 2021;22:12596. 34830479 10.3390/ijms222212596 PMC8625570

[53] Hu W, Wang Z, Zhang S, Lu X, Wu J, Yu K, et al. IQGAP1 promotes pancreatic cancer progression and epithelial-mesenchymal transition (EMT) through Wnt/β-catenin signaling. Sci Rep. 2019;9:7539. 31101875 10.1038/s41598-019-44048-y PMC6525164

[54] Liu J, Ni X, Li Y, Chen M, Chen W, Wu Y, et al. Downregulation of IQGAP1 inhibits epithelial-mesenchymal transition via the HIF1α/VEGF-A signaling pathway in gastric cancer. J Cell Biochem. 2019;120:15790–9. 31090961 10.1002/jcb.28849

[55] Toyama Y, Kontani K, Katada T, Shimada I. Conformational landscape alternations promote oncogenic activities of Ras-related C3 botulinum toxin substrate 1 as revealed by NMR. Sci Adv. 2019;5:eaav8945. 30891502 10.1126/sciadv.aav8945 PMC6415961

[56] Wu X, Ramachandran S, Lin M-CJ, Cerione RA, Erickson JW. A minimal Rac activation domain in the unconventional guanine nucleotide exchange factor Dock180. Biochemistry. 2011;50:1070–80. 21033699 10.1021/bi100971y PMC3048587

[57] Tomino T, Tajiri H, Tatsuguchi T, Shirai T, Oisaki K, Matsunaga S, et al. DOCK1 inhibition suppresses cancer cell invasion and macropinocytosis induced by self-activating Rac1P29S mutation. Biochem Biophys Res Commun. 2018;497:298–304. 29432733 10.1016/j.bbrc.2018.02.073

[58] Ji L, Xu S, Luo H, Zeng F. Insights from DOCK2 in cell function and pathophysiology. Front Mol Biosci. 2022;9:997659. 36250020 10.3389/fmolb.2022.997659 PMC9559381

[59] Hasan MK, Yu J, Widhopf GF, Rassenti LZ, Chen L, Shen Z, et al. Wnt5a induces ROR1 to recruit DOCK2 to activate Rac1/2 in chronic lymphocytic leukemia. Blood, J Am Soc Hematol. 2018;132:170–8. 10.1182/blood-2017-12-819383 PMC6043980 29678828

[60] Nishihara H, Maeda M, Oda A, Tsuda M, Sawa H, Nagashima K, et al. DOCK2 associates with CrkL and regulates Rac1 in human leukemia cell lines. Blood. 2002;100:3968–74. 12393632 10.1182/blood-2001-11-0032

[61] Wu M, Small D, Duffield AS. DOCK2: A novel FLT3/ITD leukemia drug target. Oncotarget. 2017;8:88253. 29179430 10.18632/oncotarget.21390 PMC5687600

[62] Uhlenbrock K, Eberth A, Herbrand U, Daryab N, Stege P, Meier F, et al. The RacGEF Tiam1 inhibits migration and invasion of metastatic melanoma via a novel adhesive mechanism. J Cell Sci. 2004;117:4863–71. 15340013 10.1242/jcs.01367

[63] Cool RH, Schmidt G, Lenzen CU, Prinz H, Vogt D, Wittinghofer A. The Ras mutant D119N is both dominant negative and activated. Mol Cell Biol. 1999;19:6297–305. 10.1128/mcb.19.9.6297 PMC84598 10454576

[64] Gerboth S, Frittoli E, Palamidessi A, Baltanas FC, Salek M, Rappsilber J, et al. Phosphorylation of SOS1 on tyrosine 1196 promotes its RAC GEF activity and contributes to BCR-ABL leukemogenesis. Leukemia. 2018;32:820–7. 28819285 10.1038/leu.2017.267 PMC5739283

[65] Yamahashi Y, Lin Y-H, Mouri A, Iwanaga S, Kawashima K, Tokumoto Y, et al. Phosphoproteomic of the acetylcholine pathway enables discovery of the PKC-β-PIX-Rac1-PAK cascade as a stimulatory signal for aversive learning. Mol Psychiatry. 2022;27:3479–92. 35665767 10.1038/s41380-022-01643-2 PMC9708603

[66] Feng Q, Baird D, Yoo S, Antonyak M, Cerione RA. Phosphorylation of the cool-1/β-Pix protein serves as a regulatory signal for the migration and invasive activity of Src-transformed cells. J Biol Chem. 2010;285:18806–16. 20375009 10.1074/jbc.M109.098079 PMC2881803

[67] Scita G, Tenca P, Areces LB, Tocchetti A, Frittoli E, Giardina G, et al. An effector region in Eps8 is responsible for the activation of the Rac-specific GEF activity of Sos-1 and for the proper localization of the Rac-based actin–polymerizing machine. J cell Biol. 2001;154:1031–44. 11524436 10.1083/jcb.200103146 PMC2196181

[68] Halaban R. RAC1 and melanoma. Clin Ther. 2015;37:682–5. 25465943 10.1016/j.clinthera.2014.10.027 PMC4415501

[69] Hodis E, Watson IR, Kryukov GV, Arold ST, Imielinski M, Theurillat J-P, et al. A landscape of driver mutations in melanoma. Cell. 2012;150:251–63. 22817889 10.1016/j.cell.2012.06.024 PMC3600117

[70] Mar VJ, Wong SQ, Logan A, Nguyen T, Cebon J, Kelly JW, et al. Clinical and pathological associations of the activating RAC 1 P29S mutation in primary cutaneous melanoma. Pigment Cell Melanoma Res. 2014;27:1117–25. 25043693 10.1111/pcmr.12295

[71] Araiza-Olivera D, Feng Y, Semenova G, Prudnikova TY, Rhodes J, Chernoff J. Suppression of RAC1-driven malignant melanoma by group A PAK inhibitors. Oncogene. 2018;37:944–52. 29059171 10.1038/onc.2017.400 PMC5814328

[72] Brown MD, Sacks DB. IQGAP1 in cellular signaling: bridging the GAP. Trends Cell Biol. 2006;16:242–9. 16595175 10.1016/j.tcb.2006.03.002

[73] Johnson M, Sharma M, Henderson BR. IQGAP1 regulation and roles in cancer. Cell Signal. 2009;21:1471–8. 19269319 10.1016/j.cellsig.2009.02.023

[74] Ren J-G, Li Z, Crimmins DL, Sacks DB. Self-association of IQGAP1: characterization and functional sequelae. J Biol Chem. 2005;280:34548–57. 16105843 10.1074/jbc.M507321200

[75] Marei H, Carpy A, Macek B, Malliri A. Proteomic analysis of Rac1 signaling regulation by guanine nucleotide exchange factors. Cell Cycle. 2016;15:1961–74. 27152953 10.1080/15384101.2016.1183852 PMC4968972

[76] Mirzaiebadizi A, Shafabakhsh R, Ahmadian MR. Modulating PAK1: accessory proteins as promising therapeutic targets. Biomolecules. 2025;15:242. 40001545 10.3390/biom15020242 PMC11852631

[77] Huynh N, Liu KH, Yim M, Shulkes A, Baldwin GS, He H. Demonstration and biological significance of a gastrin-P21-activated kinase 1 feedback loop in colorectal cancer cells. Physiol Rep. 2014;2:e12048. 24963032 10.14814/phy2.12048 PMC4208650

[78] Ren J-G, Li Z, Sacks DB. IQGAP1 modulates activation of B-Raf. Proc Natl Acad Sci USA. 2007;104:10465–9. 17563371 10.1073/pnas.0611308104 PMC1965536

[79] Bardwell AJ, Lagunes L, Zebarjedi R, Bardwell L. The WW domain of the scaffolding protein IQGAP1 is neither necessary nor sufficient for binding to the MAPKs ERK1 and ERK2. J Biol Chem. 2017;292:8750–61. 28396345 10.1074/jbc.M116.767087 PMC5448102

[80] Caye A, Strullu M, Guidez F, Cassinat B, Gazal S, Fenneteau O, et al. Juvenile myelomonocytic leukemia displays mutations in components of the RAS pathway and the PRC2 network. Nat Genet. 2015;47:1334–40. 26457648 10.1038/ng.3420

[81] Nishikimi A, Uruno T, Duan X, Cao Q, Okamura Y, Saitoh T, et al. Blockade of inflammatory responses by a small-molecule inhibitor of the Rac activator DOCK2. Chem Biol. 2012;19:488–97. 22520755 10.1016/j.chembiol.2012.03.008

