# Supplementary material for: New insights into the classification of the RAC1 P29S hotspot mutation in melanoma as an oncogene
Source: Cancer Gene Ther. 2025 Oct 1;32(12):1341–55. doi: 10.1038/s41417-025-00965-x (PMC12702776; doi:10.1038/s41417-025-00965-x)
Supplement: Supplementary file 2 — Original data files [file 41417_2025_965_MOESM2_ESM.pdf]

Original data files

New insights into the classification of the RAC1 P29S hotspot mutation in melanoma as an oncogene

Amin Mirzaiebadizi[1*], Mohammad Reza Ahmadian[1*]
Institute of Biochemistry and Molecular Biology II, Medical Faculty and University Hospital Düsseldorf, Heinrich Heine University, 40225 Düsseldorf, Germany

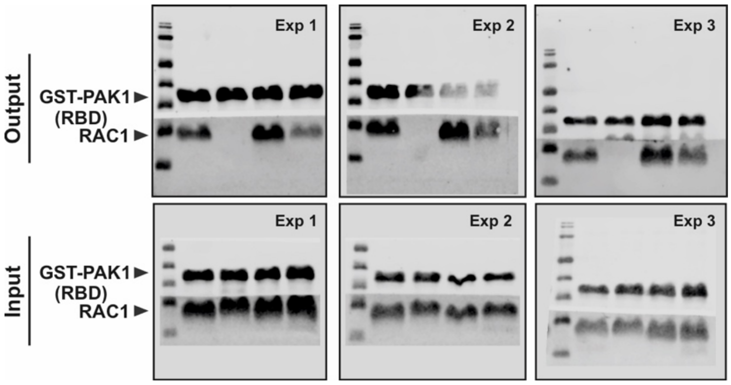

Measurements of PAK1 interaction with the RAC1 proteins. A GST pull-down assay was performed in triplicate to assess the interaction between RAC1 mutants and GST-PAK1 RBD. The top blots show the pull-down signals (output), while the bottom blots show the input samples before bead incubation. GST-PAK1 RBD was detected with an anti-GST antibody, and RAC1 proteins were visualized with an anti-RAC1 antibody. The input blots confirm that identical amounts of protein were used before bead incubation, ensuring experiment accuracy. A cropped version of experiment 1 is shown in Figure 3B, with data points and statistical analyses displayed as bar graphs in Figure 3C.

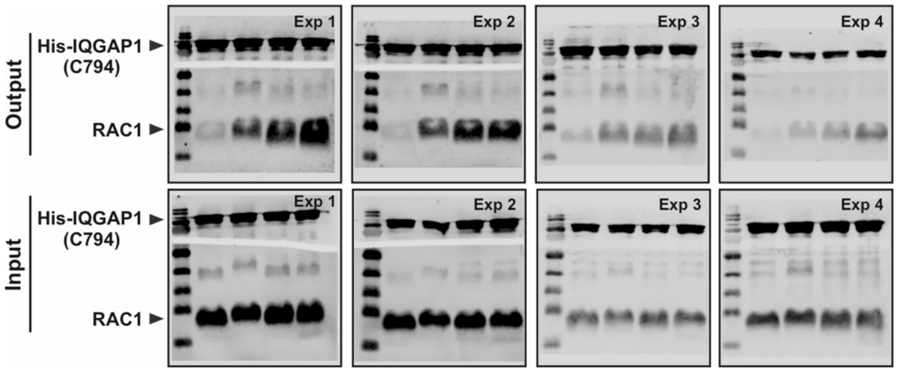

Measurements of IQGAP1 interaction with the RAC1 proteins. The His pull-down assay was performed in quadruplicate to investigate the interaction between RAC1 mutants and His-IQGAP1 C794. The top blots show the pull-down signals (output), while the bottom blots show the input samples before incubation with His-Mag-Sepharose Ni beads. His-IQGAP1 C794 was detected with an anti-His antibody, and RAC1 proteins were visualized with an anti-RAC1 antibody. The input blots confirm that identical amounts of proteins were used before bead incubation, ensuring the reliability of the results. A cropped version of Experiment 1 is shown in Figure 3H, with data points and statistical analyses presented as bar graphs in Figure 3I.

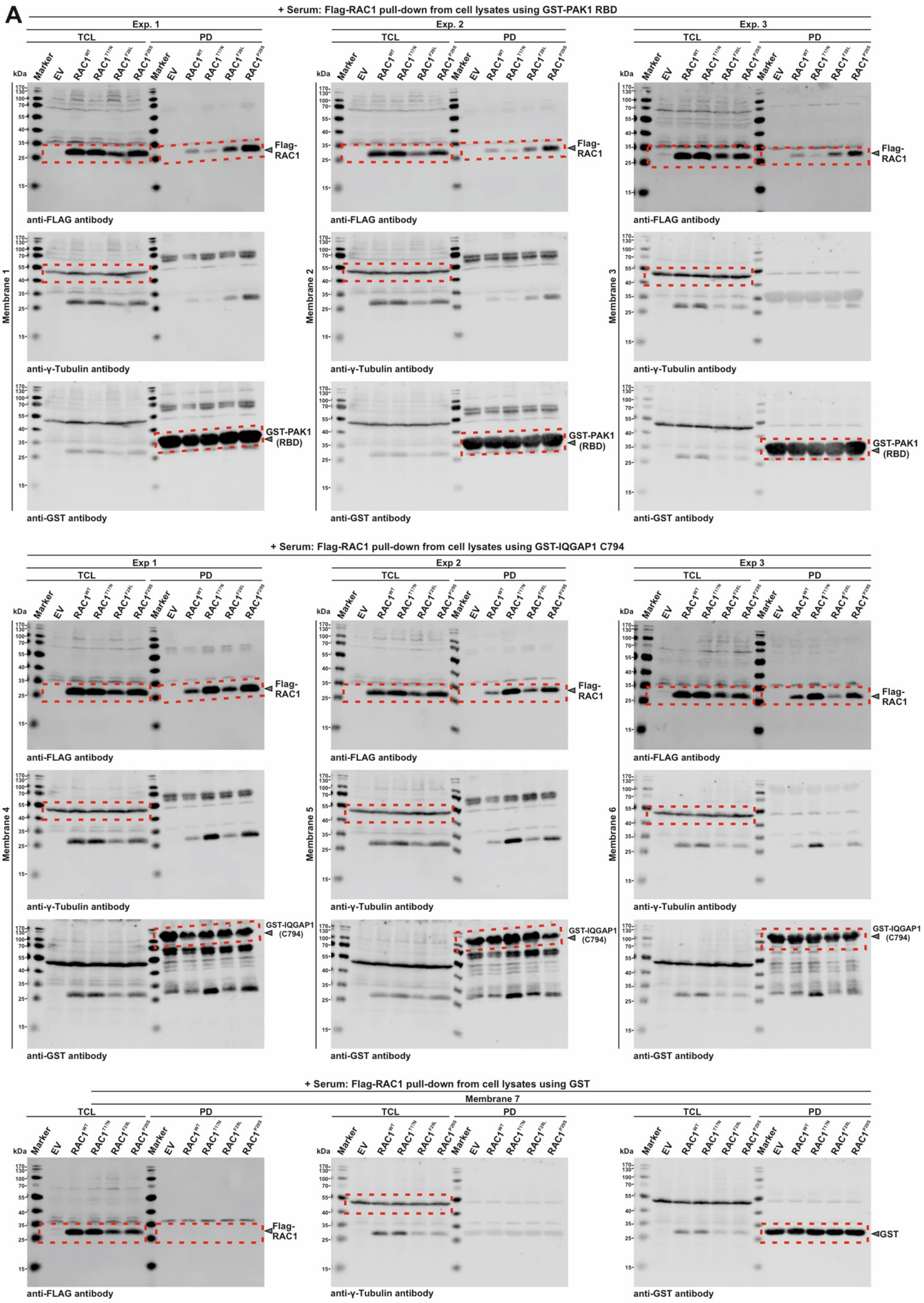
A
+ Serum: Flag-RAC1 pull-down from cell lysates using GST-PAK1 RBD
Exp. 1
Exp. 2
Exp. 3
TCL
PD
Marker
EV
RAC1WT
RAC1T17N
RAC1F28L
RAC1P29S
kDa
Flag-RAC1
anti-FLAG antibody
anti-γ-Tubulin antibody
GST-PAK1 (RBD)
anti-GST antibody
Membrane 1
Membrane 2
Membrane 3
+ Serum: Flag-RAC1 pull-down from cell lysates using GST-IQGAP1 C794
Exp 1
Exp 2
Exp 3
GST-IQGAP1 (C794)
Membrane 4
Membrane 5
Membrane 6
+ Serum: Flag-RAC1 pull-down from cell lysates using GST
Membrane 7
GST

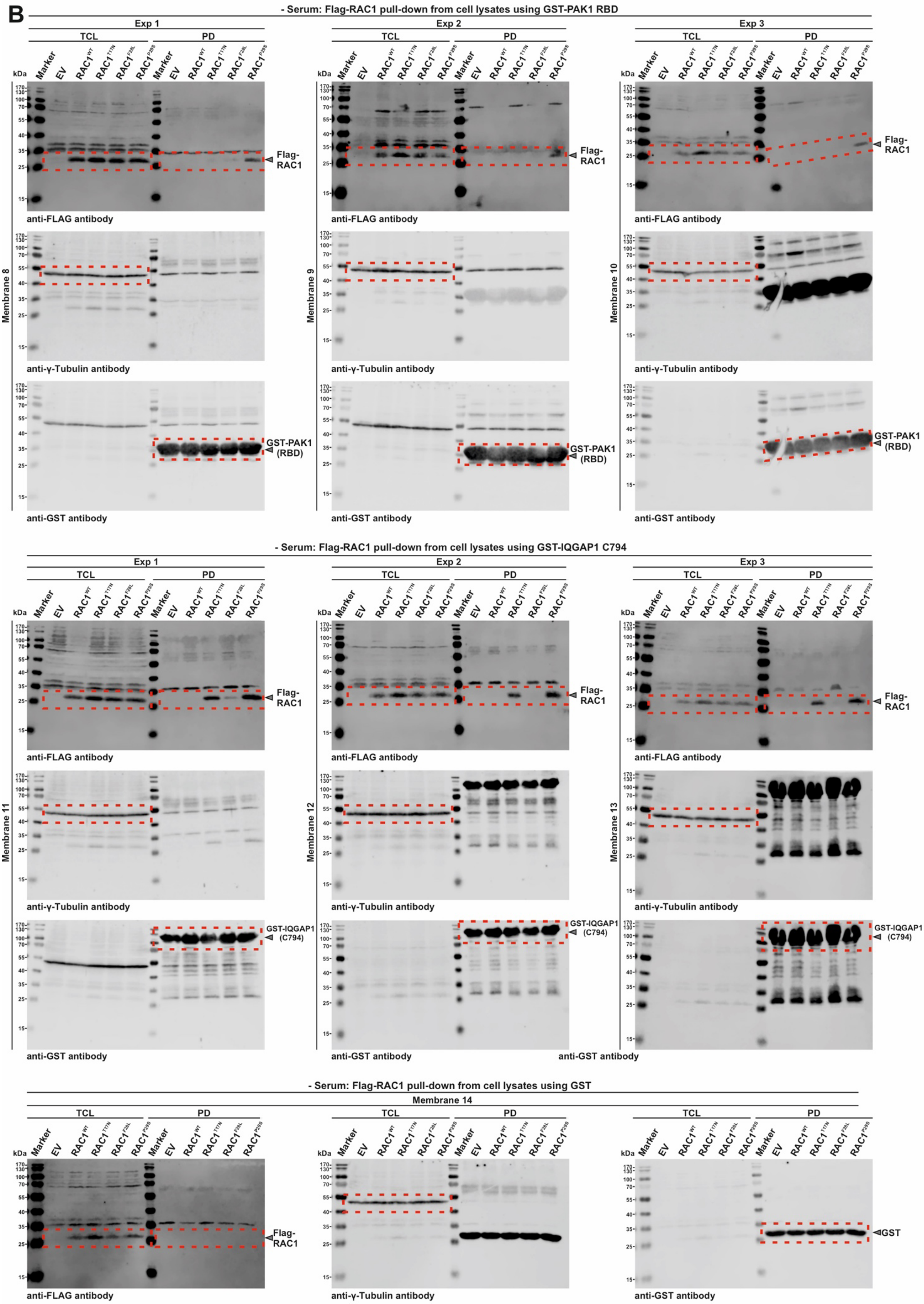
B
- Serum: Flag-RAC1 pull-down from cell lysates using GST-PAK1 RBD
Exp 1
Exp 2
Exp 3
TCL
PD
Marker
EV
RAC1WT
RAC1T17N
RAC1F28L
RAC1P29S
kDa
Flag-RAC1
anti-FLAG antibody
Membrane 8
Membrane 9
Membrane 10
anti-γ-Tubulin antibody
GST-PAK1 (RBD)
anti-GST antibody
- Serum: Flag-RAC1 pull-down from cell lysates using GST-IQGAP1 C794
Membrane 11
Membrane 12
Membrane 13
GST-IQGAP1 (C794)
- Serum: Flag-RAC1 pull-down from cell lysates using GST
Membrane 14
GST

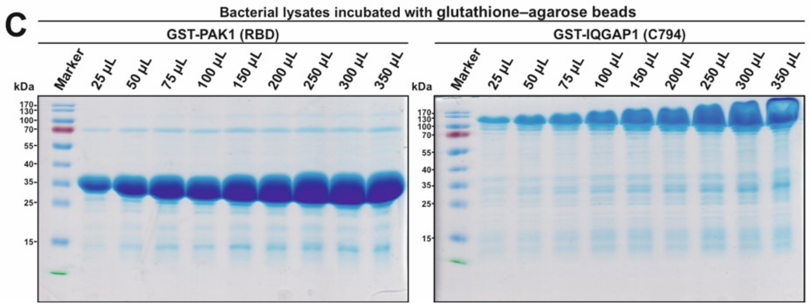

Active GTPase pull-down assays using GTP-bound Flag-RAC1 from HEK-293T cell lysates. Active GTPase pull-down assays were performed to quantify GTP-bound Flag-RAC1 from HEK-293T cell lysates with GST-PAK1 RBD, GST-IQGAP1 C794, and GST as negative controls. The assays were conducted under both serum-stimulated (**A**) and serum-starved (**B**) conditions. HEK-293T cells were transfected with RAC1 constructs, serum stimulated or starved for 24 hours, and then harvested and lysed for the pull-down assays. (**A**) shows three panels under serum-stimulated conditions (from top to bottom): the top panel represents GTP-bound Flag-RAC1 pull-down using GST-PAK1 RBD (performed in triplicate), the middle panel represents GST-IQGAP1 C794 (also in triplicate), and the bottom panel shows the negative control, GST, with no interaction observed with Flag-RAC1. (**B**) shows the same three panels under serum-starved conditions. Western blots were probed with anti-Flag, anti-GST, and anti-γ-tubulin antibodies to detect GTP-bound Flag-RAC1, the GST fusion proteins, and γ-tubulin, respectively. Molecular weights (in kDa) are indicated for each protein band. Pull-down (PD) lanes show GTP-bound Flag-RAC1 captured by the bait-bound beads, while total cell lysate (TCL) lanes show Flag-RAC1 expression levels, with γ-tubulin as a loading control. Each membrane contains samples from three independent experiments, labeled Exp1-3, with antibody incubations indicated by red dashed boxes. Membranes 1-3 represent GST-PAK1 RBD under serum stimulation; membranes 4-6, GST-IQGAP1 C794 under serum stimulation; membrane 7, GST under serum stimulation; membranes 8-10, GST-PAK1 RBD under serum starvation; membranes 11-13, GST-IQGAP1 under serum starvation; and membrane 14, GST under serum starvation. (**C**) Coomassie-stained SDS-PAGE Gels showing the bead saturation assay for GST-PAK1 RBD (left) and GST-IQGAP1 C794 (right). After IPTG induction and protein expression, varying amounts of bacterial lysate (25-350 µL) were incubated with 50 µL of GSH beads for one hour and washed three times. The beads were then mixed with SDS-Laemmli sample buffer, heated to 95°C, and analyzed by SDS-PAGE fallowed by Coomassie Brilliant Blue staining. Based on this pre-test, 25 µL of GST-PAK1 RBD lysate and 50 µL of GST-IQGAP1 C794 lysate were used for the active GTPase pull-down assays.

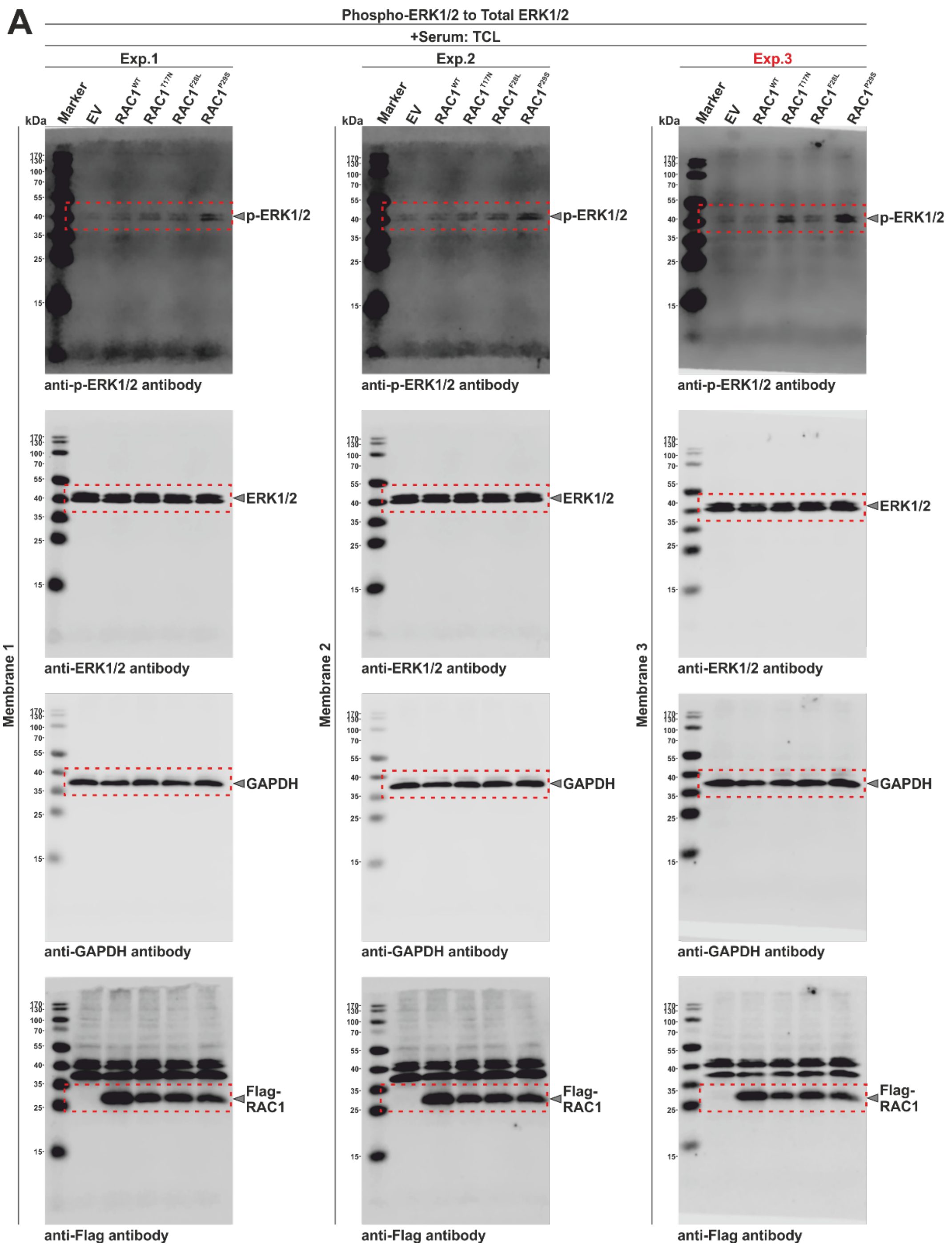
A
Phospho-ERK1/2 to Total ERK1/2
+Serum: TCL
Exp.1
Exp.2
Exp.3
kDa
Marker
EV
RAC1WT
RAC1T17N
RAC1F28L
RAC1P29S
p-ERK1/2
anti-p-ERK1/2 antibody
ERK1/2
anti-ERK1/2 antibody
GAPDH
anti-GAPDH antibody
Flag-RAC1
anti-Flag antibody
Membrane 1
Membrane 2
Membrane 3

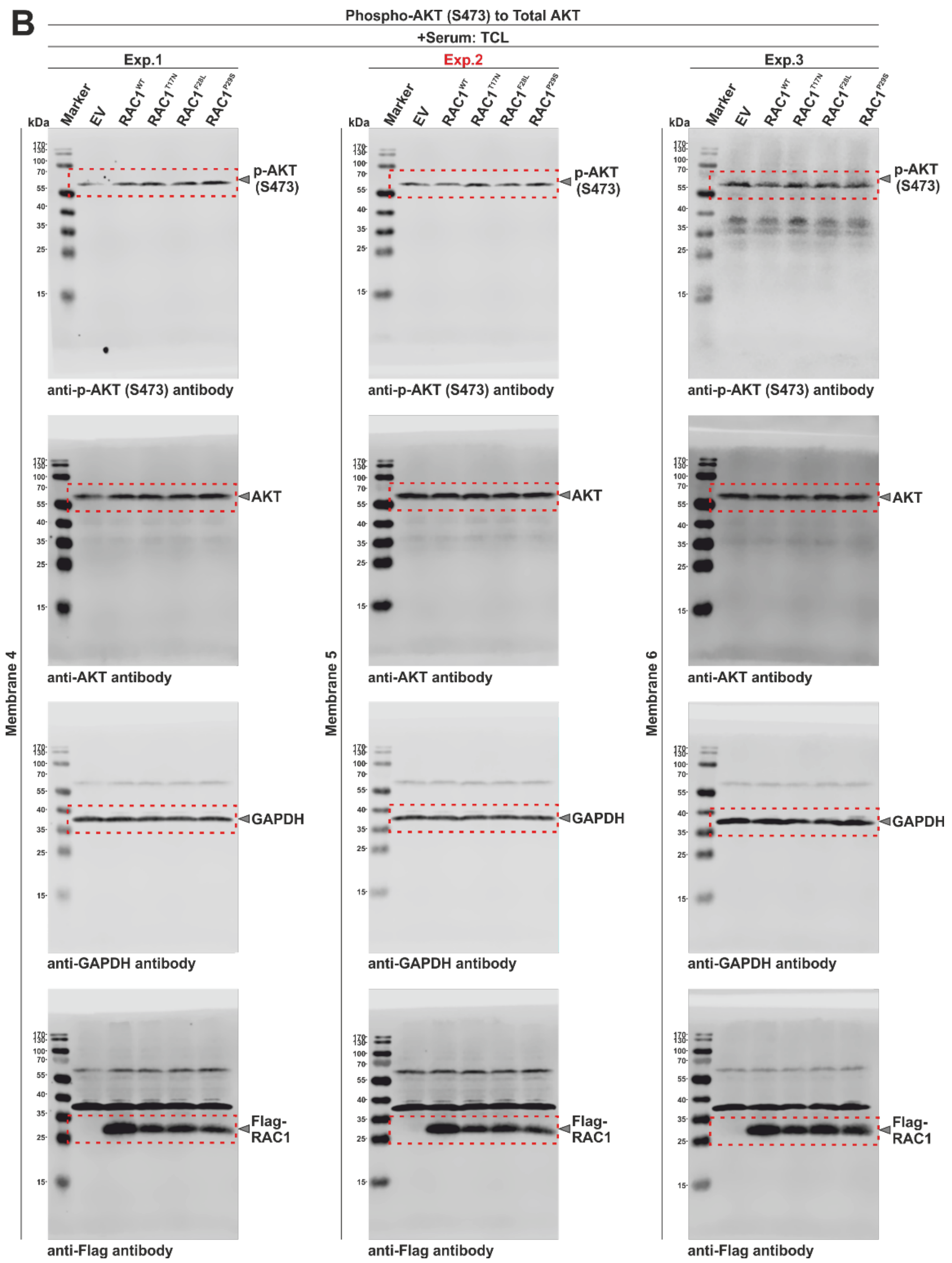
B
Phospho-AKT (S473) to Total AKT
+Serum: TCL
Exp.1
Exp.2
Exp.3
Marker
EV
kDa
p-AKT (S473)
anti-p-AKT (S473) antibody
AKT
anti-AKT antibody
GAPDH
anti-GAPDH antibody
Flag-RAC1
anti-Flag antibody
Membrane 4
Membrane 5
Membrane 6

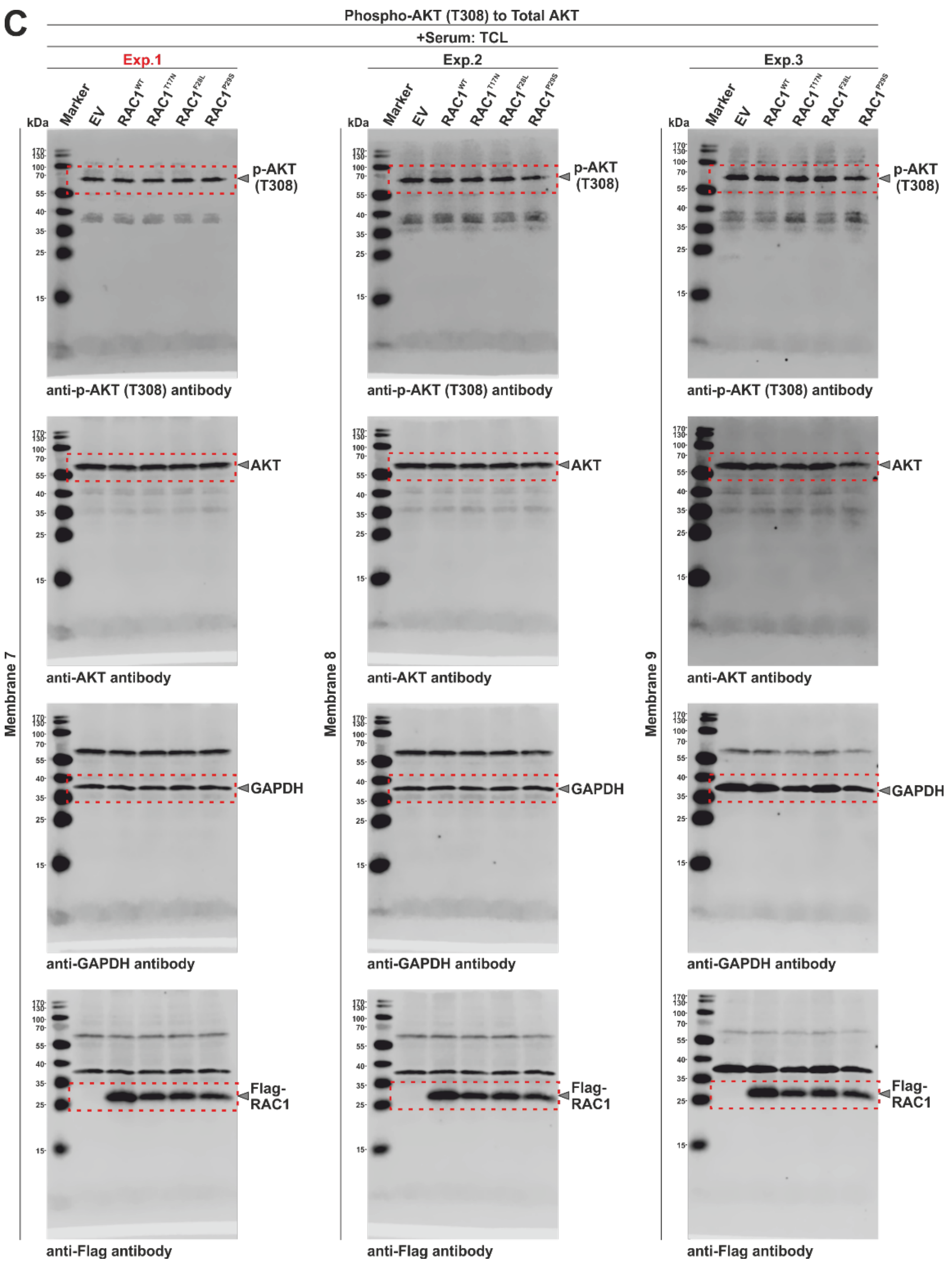
C
Phospho-AKT (T308) to Total AKT
+Serum: TCL
Exp.1
Exp.2
Exp.3
Marker
EV
RAC1WT
RAC1T17N
RAC1F28L
RAC1P29S
kDa
p-AKT (T308)
AKT
GAPDH
Flag-RAC1
anti-p-AKT (T308) antibody
anti-AKT antibody
anti-GAPDH antibody
anti-Flag antibody
Membrane 7
Membrane 8
Membrane 9

D

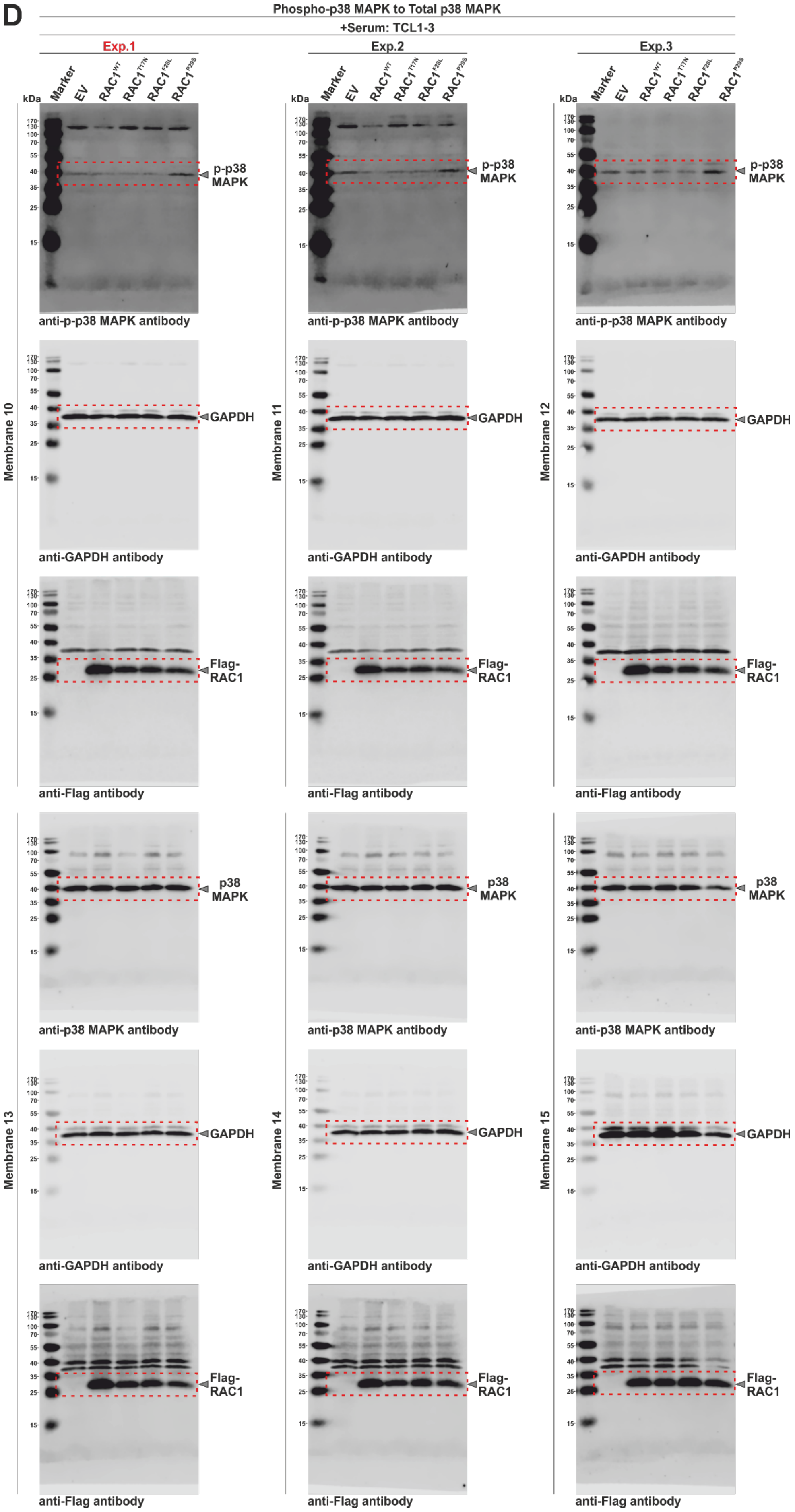
Phospho-p38 MAPK to Total p38 MAPK
+Serum: TCL1-3
Exp.1
Exp.2
Exp.3
kDa
Marker
EV
RAC1WT
RAC1T17N
RAC1F28L
RAC1P29S
p-p38 MAPK
GAPDH
Flag-RAC1
p38 MAPK
Membrane 10
Membrane 11
Membrane 12
Membrane 13
Membrane 14
Membrane 15
anti-p-p38 MAPK antibody
anti-GAPDH antibody
anti-Flag antibody
anti-p38 MAPK antibody

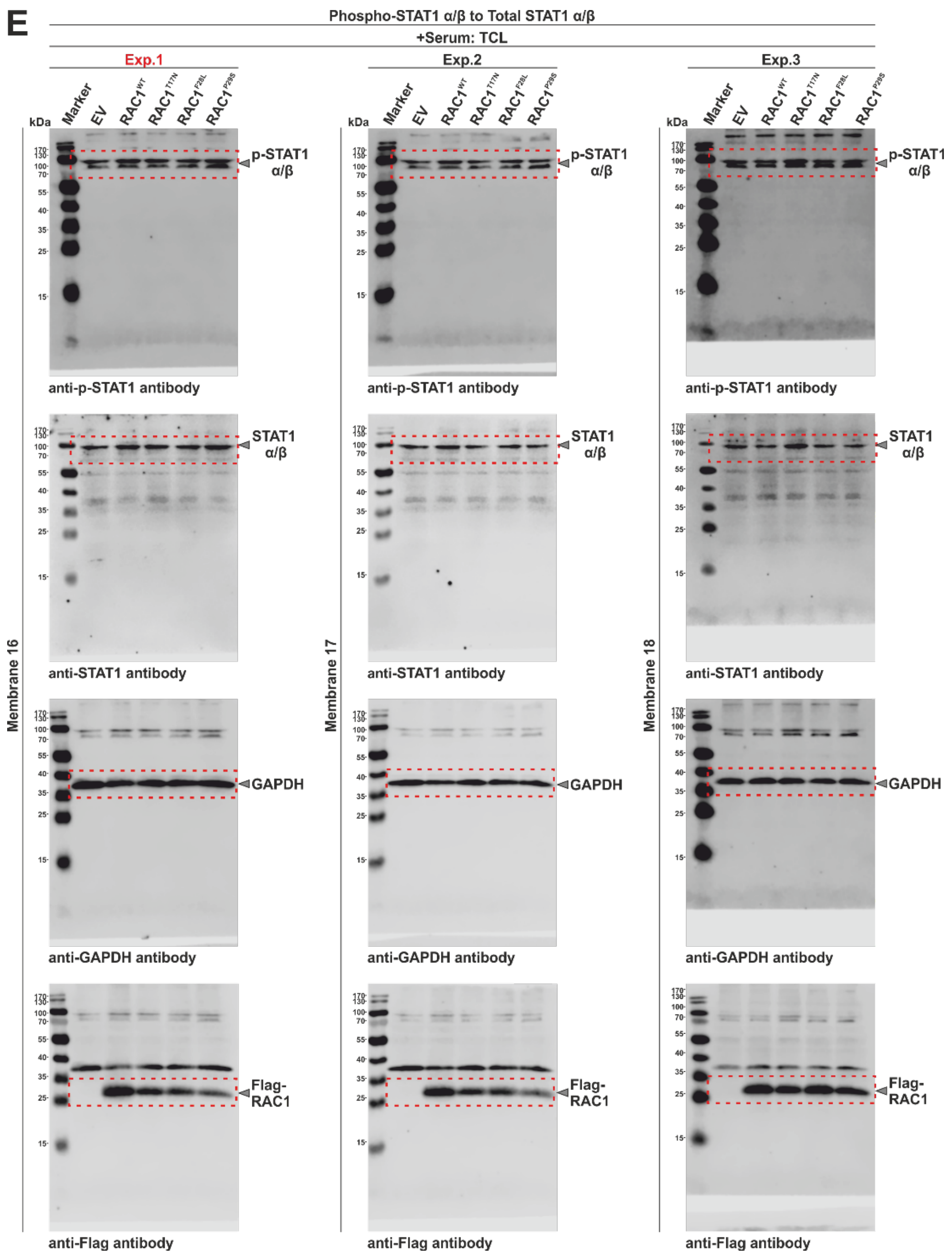

Western blotting and phosphorylation analysis of ERK1/2, AKT(S473), AKT(T308), p38 MAPK, and STAT1 α/β in HEK-293T cells overexpressing RAC1 variants. Western blot analysis was performed on serum-stimulated HEK-293T cells transiently transfected with pcDNA3.1 constructs encoding Flag-tagged RAC1 variants (WT, T17N, F28L, and P29S) alongside an empty vector (EV) control. Experiments were performed in triplicate, with each experiment visualized on nitrocellulose membranes and labeled as follows: (A) phospho-ERK1/2 to total ERK1/2 (membranes 1-3); (B) phospho-AKT (S473) to total AKT (membranes 4-6); (C) phospho-AKT (T308) to total AKT (membranes 7-9); (D) phospho-p38 MAPK to total p38 MAPK (membranes 10-15); and (E) phospho-STAT1 α/β to total STAT1 α/β (membranes 16-18). Cropped sections of the membrane shown in Figure 5 are as follows: Exp1 for AKT(T308), p38 MAPK, and STAT1 α/β; Exp2 for AKT(S473); Exp3 for ERK. Each membrane was probed sequentially, starting with the respective anti-phospho antibodies, followed by antibodies against ERK, AKT, p38 MAPK, STAT1 α/β, and GAPDH as loading controls, and the flag antibody to validate RAC1 variant expression. For p38 MAPK, separate membranes were used to detect phosphorylated and total form, as both antibodies are derived from the same host species. Detected protein bands are highlighted with dashed red boxes, and arrows indicate the corresponding molecular weight in kDa for each protein. Abbreviations: TCL = total

cell lysate; Serum+ = serum-stimulated HEK-293T cells. See the Materials and Methods section for further details.

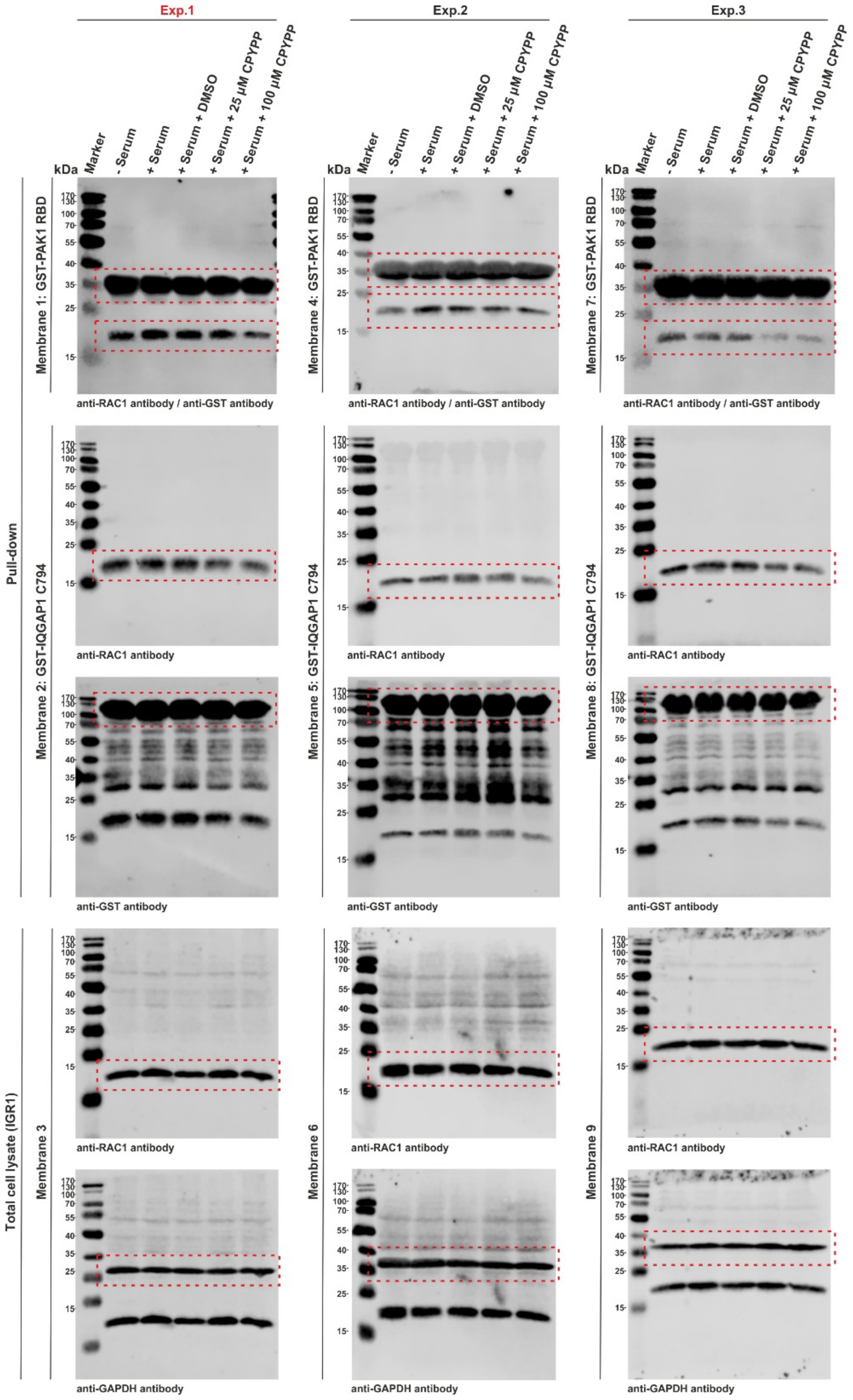

Original Western blot data from active GTPase pull-down assays detecting GTP-bound RAC1^{P29S} in IGR1 human melanoma cells under various treatment conditions. IGR1 cells that endogenously express the RAC1^{P29S} mutant were cultured under either serum-stimulated or serum-starved conditions. These cells were then treated for three hours with 0.5 % DMSO and with 25 or 100 µM CPYPP, which is a DOCK2 inhibitor. GTPase pull-downs were performed using glutathione agarose beads coupled to GST-tagged PAK1 RBD or IQGAP1 C794 to isolate active RAC1^{P29S}•GTP. The eluted protein complexes were resolved by SDS-PAGE and probed with anti-RAC1, anti-GST, and anti-GAPDH antibodies to detect active RAC1^{P29S}, the bait proteins, and the loading control, respectively. Pull-down and total cell lysate lanes are shown for each condition. The figure displays original, uncropped blots from three biological replicates. Quantified data derived from these blots are presented in Figures 6B and 6C of the main manuscript.
